# Variation in microparasite free-living survival and indirect transmission can modulate the intensity of emerging outbreaks

**DOI:** 10.1038/s41598-020-77048-4

**Published:** 2020-11-27

**Authors:** C. Brandon Ogbunugafor, Miles D. Miller-Dickson, Victor A. Meszaros, Lourdes M. Gomez, Anarina L. Murillo, Samuel V. Scarpino

**Affiliations:** 1 Department of Ecology and Evolutionary Biology, Yale University, New Haven, CT 06511 USA; 2 Department of Ecology and Evolutionary Biology, Brown University, Providence, 02912 USA; 3 Center for Computational Molecular Biology, Brown University, Providence, 02912 USA; 4 Department of Pediatrics, Warren Alpert Medical School, Brown University, Providence, 02912 USA; 5 Center for Statistical Sciences, Brown University School of Public Health, Providence, 02903 USA; 6 Network Science Institute, Northeastern University, Boston, 02115 USA; 7 Roux Institute, Northeastern University, Portland, 04101 USA; 8 Santa Fe Institute, Santa Fe, 87501 USA

**Keywords:** Dynamical systems, Infectious diseases, Ecological epidemiology

## Abstract

Variation in free-living microparasite survival can have a meaningful impact on the ecological dynamics of established and emerging infectious diseases. Nevertheless, resolving the importance of indirect and environmental transmission in the ecology of epidemics remains a persistent challenge. It requires accurately measuring the free-living survival of pathogens across reservoirs of various kinds and quantifying the extent to which interaction between hosts and reservoirs generates new infections. These questions are especially salient for emerging pathogens, where sparse and noisy data can obfuscate the relative contribution of different infection routes. In this study, we develop a mechanistic, mathematical model that permits both direct (host-to-host) and indirect (environmental) transmission and then fit this model to empirical data from 17 countries affected by an emerging virus (SARS-CoV-2). From an ecological perspective, our model highlights the potential for environmental transmission to drive complex, nonlinear dynamics during infectious disease outbreaks. Summarizing, we propose that fitting alternative models with indirect transmission to real outbreak data from SARS-CoV-2 can be useful, as it highlights that indirect mechanisms may play an underappreciated role in the dynamics of infectious diseases, with implications for public health.

## Introduction

The ecology of infectious disease has provided a theoretical basis for understanding how interactions between microbes, their hosts, and environments shape the severity of epidemics. Mathematical modeling methods offer a means of exploring the consequences of different routes of transmission on the shape of epidemics. This is useful in the case of emerging diseases, where it may be challenging to fully resolve all of the features of an epidemic, including the influence of different routes of transmission on key features of an epidemic. That is, even though there is a large general literature on environmental transmission (or “fomite” transmission)^1–13^, relatively few studies have explored how particular aspects of indirect or environmental transmission can influence disease dynamics. Lack of clarity regarding how variation in environmental transmission (e.g. as driven by different physical surfaces) manifests in epidemics is especially notable in emerging diseases, where data sets are elusive, sparse, or noisy.

For example, Severe Acute Respiratory Syndrome Coronavirus 2 (SARS-CoV-2), the etiological agent of coronavirus disease 2019 (COVID-19), has caused one of the largest pandemics of the last century. The complex set of epidemiological characteristics defining COVID-19 outbreaks presents a number of challenges for controlling this disease. As a consequence, countries have achieved varying levels of success^14–18^ in reducing transmission and protecting vulnerable populations, often with dramatic variation from setting to setting in the intensity of epidemics. The basic reproductive number (${\mathcal{R}}_{0}$)^19–21^, fatality rate^22,23^, incubation period^22,24–26^, transmission interval^27^, prevalence of super-spreading events^28,29^, and other relevant aspects of COVID-19 epidemiology provide a mechanistic window into how SARS-CoV-2 is transmitted in different settings. However, one feature of SARS-CoV-2 transmission that was validated in laboratory settings, but whose epidemiological role remains highly controversial, is SARS-CoV-2 free-living survival. Specifically, several laboratory and epidemiological findings offer conflicting evidence that indirect or environmental transmission (via free-living virus) may play a role in some settings^30–41^, and to our knowledge, none have fully investigated how this route of transmission may influence features of outbreaks. Several unknowns notwithstanding, the availability of SARS-CoV-2 data make it a valuable model for examining the particulars of indirect transmission in emerging outbreaks.

In this study, we use confirmed case data alongside laboratory and epidemiologically validated parameters to develop a mechanistic model that includes indirect transmission. We evaluate the potential for variability in indirect or environmentally-mediated transmission to explain variability in various features associated with the intensity of outbreaks. This framework includes parameters corresponding to the transmission of the virus from both presymptomatic/asymptomatic and clinical (symptomatic) carriers of virus, and the possibility that susceptible hosts can acquire infection through environmental reservoirs or other indirect means. We examine how outbreak dynamics can be influenced by differences in viral free-living survival that include empirical values for survival on various abiotic reservoirs (e.g. aerosols, plastic, copper, steel, cardboard)^30^.

Our findings highlight the need for an empirically-informed, mechanistic understanding of the ecology of emerging viral outbreaks, including the particulars of free-living survival on different abiotic surfaces and in aerosol form. These routes should be a greater focus in emerging infectious disease outbreaks, as a refined grasp on their specifics can uncover important details of outbreak dynamics.

## Materials and methods

### A waterborne, abiotic, and other indirectly transmitted (WAIT) model for the dynamics of emergent viral outbreaks

Several models have been engineered to explore aspects of COVID-19 dynamics. For example, models have been used to investigate the role of social distancing^20,42^, social mixing^43^, the importance of undocumented infections^44^, the role of mobility in the early spread of disease in China^45^, and the potential for contact tracing as a solution^46^. Only a few notable models of SARS-CoV-2 transmission incorporate features of indirect or environmental transmission^40,46^ and none consider the dynamical properties of viral free-living survival in the environment. Such a model structure would provide an avenue towards exploring how variation in free-living survival influences disease outbreaks. Indirect transmission includes those routes where pathogen is spread through means other than from person to person, and includes transmission through environmental reservoirs. Environmental transmission models are aplenty in the literature and serve as a theoretical foundation for exploring similar concepts in newer, emerging viruses^1–10^.

Here, we parameterize and validate an SEIR-W model: Susceptible (*S*), Exposed (*E*), Infectious (*I*), Recovered (*R*), and WAIT (*W*) model. Here *W* represents the environmental component of the transmission cycle during the early stage of the SARS CoV-2 pandemic. This component introduces more opportunities for infection, and complex dynamics resulting from viral persistence in the environment. In this framework, both indirect and direct transmission occur via mass-action, “random” encounters.

This model is derived from a previously developed framework called “WAIT”—which stands for *Waterborne, Abiotic,* and other *Indirectly Transmitted*—that incorporates an environmental reservoir where a pathogen remains in the environment and “waits” for hosts to interact with it^11,12^. The supplementary information contains a much more rigorous discussion of the modeling details. In the main text, we provide select details.

### Building the SEIR-W model framework for SARS-CoV-2

Here *W* represents the environmental component of the early stage of the SARS CoV-2 pandemic (Fig. S1). This environmental compartment refers to reservoirs that people may have contact with on a daily basis, such as doorknobs, appliances, and non-circulating air indoors. The *W* compartment of our model represents the fraction of these environmental reservoirs that house some sufficiently transmissible amount of infectious virus. We emphasize that the *W* compartment is meant to only represent reservoirs that are common sites for interaction with people. Thus, inclusion of the *W* compartment allows us to investigate the degree to which the environment is infectious at any given point, and its impact on the transmission dynamics of SARS CoV-2.

Model parameters are described in detail in Table 1. The system of equations in the proposed mathematical model corresponding to these dynamics are defined in Eqs. (1)–(6):

**Table 1:** Model population definitions and initial values denoted with subscript 0 for each state variable.

Symbols	Initial values	Units	Definitions	Sources
*S*_*0*_	Varies by country	People	Susceptible individuals	Vary
*E*_*0*_	${\mathcal{R}}_{0}$* · (I*_*A0*_ + *I*_*S0*_*)*	People	Exposed individuals	Deduced
*I*_*A0*_	*I*_*S0*_	People	Asymptomatic individuals	Deduced
*I*_*S0*_	Varies	People	Symptomatic individuals	Deduced
*Rec*_*0*_	0	People	Recovered individuals	Deduced
*W*_*0*_	1%	Unitless	% of viruses in environment	Deduced

Here we present definitions for the population groups represented by each compartment as well as their initial values. The initial value of the *S* and *I*_*S*_ populations vary by country, as shown in Table 2. We take the initial value of the *I*_*A*_ population to be the same as the initial value of symptomatic individuals as a conservative estimate. The initial value of the *E* population is computed by assuming that all initially-infected people (*I*_*A0*_ + *I*_*S0*_) have exposed the virus to approximately other people.

$${\mathcal{R}}_{0}$$ (≈ 2.5).

1 $$\frac{dS}{dt}=\mu (N-S)-\left(\frac{{\beta }_{A}{I}_{A}+{\beta }_{S}{I}_{S}}{N}+{\beta }_{W}W\right)S$$

2 $$\frac{dE}{dt}=\left(\frac{{\beta }_{A}{I}_{A}+{\beta }_{S}{I}_{S}}{N}+{\beta }_{W}W\right)S-(\epsilon +\mu )E$$

3 $$\frac{d{I}_{A}}{dt}=\epsilon E-(\omega +\mu ){I}_{A}$$

4 $$\frac{d{I}_{S}}{dt}=(1-p)\omega {I}_{A}-(\nu +{\mu }_{S}){I}_{S}$$

5 $$\frac{dR}{dt}=p\omega {I}_{A}+\nu {I}_{S}-\mu R$$

6 $$\frac{dW}{dt}=\left(\frac{{\sigma }_{A}{I}_{A}+{\sigma }_{S}{I}_{S}}{N}\right)\left(1-W\right)-kW$$

### Infection trajectories

In addition to including a compartment for the environment (W), our model also deviates from traditional SEIR form by splitting the infectious compartment into an *I*_*A*_*-*compartment (*A* for asymptomatic), and an *I*_*S*_*-*compartment (*S* for symptomatic). As we discuss below, including asymptomatic (or sub-clinical) transmission is both essential for understanding how environmental—as opposed to simply unobserved or hidden—transmission affects the ecological dynamics of pathogens and also for analyzing SARS-CoV-2. The former represents an initial infectious stage (following the non-infectious, exposed stage), from which individuals will either move on to recovery directly (representing those individuals who experienced mild to no symptoms) or move on to the *I*_*S*_*-*compartment (representing those with a more severe response). Finally, individuals in the *I*_*S*_*-*compartment will either move on to recovery or death due to the infection. This splitting of the traditional infectious compartment is motivated by mounting evidence of asymptomatic transmission of SARS CoV-2^44,47–50^. Thus, we consider two trajectories for the course of the disease, similar to those employed in prior studies^42^: (1) *E → I*_*A*_* → R* and (2) *E → I*_*A*_* → I*_*S*_* → R (or death)*. More precisely, once in the *E* state, an individual will transition to the infectious state *I*_*A*_, at a per-person rate of ε. A proportion *p* will move from *I*_*A*_ to the recovered state *R* (at a rate of *p* ⍵). A proportion *(1—p)* of individuals in the *I*_*A*_ state will develop more severe systems and transition to *I*_*s*_ (at a rate of *(1—p)* ⍵). Individuals in the *I*_*s*_ state recover at a per-person rate of ν or die at a per-person rate *μ*_*S*_. In each state, normal mortality of the individual occurs at the per-person rate *μ* and newly susceptible (*S*) individuals enter the population at a rate *μN*. The important differences between these two trajectories are in how likely an individual is to move down one path or another, how infectious individuals are (both for people and for the environment), how long individuals spend in each trajectory, and how likely death is along each trajectory.

### Clarification on the interactions between hosts and reservoirs

The model couples the environment and people in two ways: (1) people can deposit the infectious virus onto environmental reservoirs (e.g. physical surfaces, and in the case of aerosols, the ambient air) and (2) people can become infected by interacting with these reservoirs. While most of our study is focused on physical surfaces, we also include data and analysis of SARS-CoV-2 survival in aerosols. While aerosols likely play a more significant role in person-to-person transmission, they also facilitate an indirect means of transmitting. For example, because SARS-CoV-2 can remain suspended in the air, other individuals can become infected without ever having to be in especially close physical proximity to the aerosol emitter (only requires that they interact with the same stagnant air, containing infectious aerosol particles)^51^. That is, a hypothetical infectious person A may produce aerosols, leave a setting, and those aerosols may infect a susceptible individual B who was never in close proximity to person A. In the transmission event between person A and person B, aerosol transmission functions in a similar fashion to surface transmission, where aerosols may be exchanged in the same room where infected individuals were, rather than exchanging infectious particles on a surface.

In our model, indirect infection via aerosols is encoded into the terms associated with the *W* component, just as the different physical surfaces are. Alternatively, aerosol transmission that leads to direct infection between hosts is encoded in the terms associated with direct infection between susceptible individuals and those infected (see section entitled Infection Trajectories).

Environmental reservoirs infect people through the *β*_*W*_ term (Eqs. 1 and 2), a proxy for a standard transmission coefficient, corresponding specifically to the probability of successful infectious transmission from the environment reservoir to a susceptible individual (the full rate term being *β*_*W*_*W·S*). Hence, the *β*_*W*_ factor is defined as the fraction of people who interact with the environment daily, per fraction of the environment, times the probability of transmitting infection from environmental reservoir to people. The factor *β*_*W*_*W* (where *W* is the fraction of environmental reservoirs infected) represents the daily fraction of people that will interact with the infected portion of the environment and become infected themselves. The full term *β*_*W*_*W·S* is thus the total number of infections caused by the environment per day.

In an analogous manner, we model the spread of infection *to* the environment with the two terms σ_*A*_* I*_*A*_*·(1—W) / N* and σ_*S*_* I*_*S*_*·(1—W) / N* representing deposition of infection to the environment by asymptomatic individuals, in the former, and symptomatic individuals, in the latter. In this case, σ_*A*_ (and analogously for σ_S_) gives the fraction of surfaces/reservoirs that interact with people at least once per day, times the probability that a person (depending on whether they are in the *I*_*A*_ or the *I*_*S*_ compartment) will deposit an infectious viral load to the reservoir. Thus, σ_*A*_* I*_*A*_* / N* and σ_*S*_* I*_*S*_* / N* (where *N* is the total population of people) represent the daily fraction of the environment that interacts with asymptomatic and symptomatic individuals, respectively. Lastly, the additional factor of *(1—W)* gives the fraction of reservoirs in the environment that have the potential for becoming infected, and so σ_*A*_* I*_*A*_*·(1—W) / N* (and analogously for *I*_*S*_) gives the fraction of the environment that becomes infected by people each day. We use *W* to represent a fraction of the environment, although one could also have multiplied the *W* equation by a value representing the total number of reservoirs in the environment (expected to remain constant throughout the course of the epidemic, assuming no intervention strategies).

### Parameter values estimation

Table 1 displays information on the population definitions and initial values in the model**.** Tables 2 and 3 contain the fixed and estimated values and their sources (respectively). The model’s estimated parameters are based on model fits to 17 countries with the highest cumulative COVID-19 cases (of the 181 total countries affected) as of 03/30/2020, who have endured outbreaks that had developed for at least 30 days following the first day with ≥ 10 cumulative infected cases within each country^14^ (See supplementary information Tables S1–S3). In addition, we compare country fits of the SEIR-W model to fits with a standard SEIR model. Lastly, we compare how various iterations of these mathematical models compare to one another with regards to the general model dynamics. For additional details, see the supplementary information.

**Table 2:** Fixed parameter values estimated based on available published literature.

Symbols	Values	Units	Definitions	Sources
μ	1/(80.3 * 365)	1/day	Natural Death Rate (Reciprocal of the average life expectancy of 17 countries sampled)	^14,65^
μ_S_	0.00159	1/day	Infected death rate	^22,66^
η	5.5	days	Incubation Period	^67^
1/⍵	η−ε^−1^	days	Expected time in the asymptomatic state	Fitted and dependent on η
ν	0.031	1/day	Recovery rate (Average of 3–6 weeks)	^67^
*p*	0.956	Unitless	Fraction that move along the “mild” recovery track	^20^
*k*	0.649	1/day	Viral decay rate in environment (using average of all material values, wood, steal, cardboard, plastic)	^30^

These estimated values derived from the existing COVID-19 and SARS-CoV-2 literature.

**Table 3:** Estimated parameter values, averaged across countries.

Symbols	Average values (SEIR-W)	SD (SEIR-W)	Average values (SEIR)	SD (SEIR)	Units	Definitions
*β*_*A*_	0.550	0.345	0.429	0.751	1/day	(Contact rate of people with people) × (transmission probability of people to people by an asymptomatic person)
*β*_*S*_	0.491	1.260	8.019	5.972	1/day	(Contact rate of people with people) × (transmission probability of people to people by *I*-person)
*β*_*W*_	0.031	0.039	0.0	–	1/day	(Contact rate of person with environment) × (transmission probability of environment to people)
σ_*A*_	3.404	6.662	0.0	–	1/day	(Contact rate of person with environment) × (probability of shedding by asymp.-person to environment)
σ_*S*_	13.492	18.849	0.0	–	1/day	(Contact rate of person with environment) × (probability of shedding by symp.-person to environment)
*1/ε*	2.478	1.325	2.381	2.249	days	Average number of days before infectious

Here we provide a table of the average values of the fitted parameters used in this model. These averages are taken across all of the selected 17 countries. See supplementary information for more details on country data and parameter estimation.

### Basic reproductive ratios (${\mathcal{R}}_{0}$)

We can express the ${\mathcal{R}}_{0}$ (Eq. 7) in a form that makes explicit the contributions from the environment and from person-to-person interactions. In this way, the full ${\mathcal{R}}_{0}$ is observed to comprise two ${\mathcal{R}}_{0}$ sub-components: one the number of secondary infections caused by a single infected person through person-to-person contact alone (*R*_*p*_) and the other is the number of secondary infections caused by exchanging infection with the environment (*R*_*e*_).

7 $${R}_{0}=\frac{{R}_{p} + \sqrt{{R}_{p}^{2} + 4 {R}_{e}^{2}}}{2}$$

where *R*_*p*_ and *R*_*e*_ are defined in Eqs. (8a) and (8b)

8a $${R}_{p}=\frac{\varepsilon ({\beta }_{A} ({\mu }_{S }+ \nu ) + {\beta }_{S}(1 - p) \omega )}{(\mu + \varepsilon )(\mu + \omega )({\mu }_{S} + \nu )}$$

8b $${R}_{e}^{2}=\frac{\varepsilon { \beta }_{W} ({\sigma }_{A} ({\mu }_{S }+ \nu ) + {\sigma }_{S}(1 - p) \omega )}{k (\mu + \varepsilon )(\mu + \omega )({\mu }_{S} + \nu )}$$

Note that when *R*_*p*_ = 0, the ${\mathcal{R}}_{0}$ reduces to *R*_*e*_ and when *R*_*e*_ = 0, the ${\mathcal{R}}_{0}$ reduces to *R*_*p*_. Thus, when person-to-person transmission is set to zero, the ${\mathcal{R}}_{0}$ consists only of terms associated with transmission from the environment, and when transmission from the environment is set to zero, the ${\mathcal{R}}_{0}$ consists only of infection directly between people. When both routes of transmission are turned on, the two ${\mathcal{R}}_{0}$*-*components combine in the manner in Eq. (7).

While *R*_*e*_ represents the component of the ${\mathcal{R}}_{0}$ formula associated with infection from the environment, the square of this quantity *R*_*e*_^*2*^ represents the expected number of *people* who become infected in the two-step infection process: people → environment → people, representing the flow of infection from people to the environment, and then from the environment to people. Thus, while *R*_*p*_ gives the expected number of people infected by a single infected person when the environmental transmission is turned off, *R*_*e*_^*2*^ gives the expected number of *people* infected by a single infected person by way of the environmental route exclusively, with no direct person-to-person transmission. Also note that *R*_*e*_^*2*^*/*(*R*_*e*_^*2*^ + *R*_*p*_) can be used to measure the extent of transmission that is mediated by the environment exclusively. This proportion can be used as a proxy for how important environmental transmission is in a given setting. Elaboration on formulas 8a–b—and associated derivation-discussions—appear in the supplementary information.

## Results

The results section covers the following sets of tests and analyses:

1. The process through which parameters were estimated through fits to country-level outbreak data.
2. Sensitivity analysis, to discuss how variation in parameters influences key aspects of virus transmission dynamics.
3. An examination of how the mathematical model incorporates indirect and environmental transmission. We discuss a calculation of the proportion of the transmission in a given setting that can be attributed to environmental transmission.
4. Simulations of “reservoir world” scenarios, where the environmental transmission value is set to one of the reservoirs for which there are published findings^30^. This is designed to identify how hypothetical settings comprising SARS-CoV-2 environmental transmission of a certain kind influences disease dynamics.

### Establishing features of indirect or environmental transmission using country outbreak data

**Figure 1:**
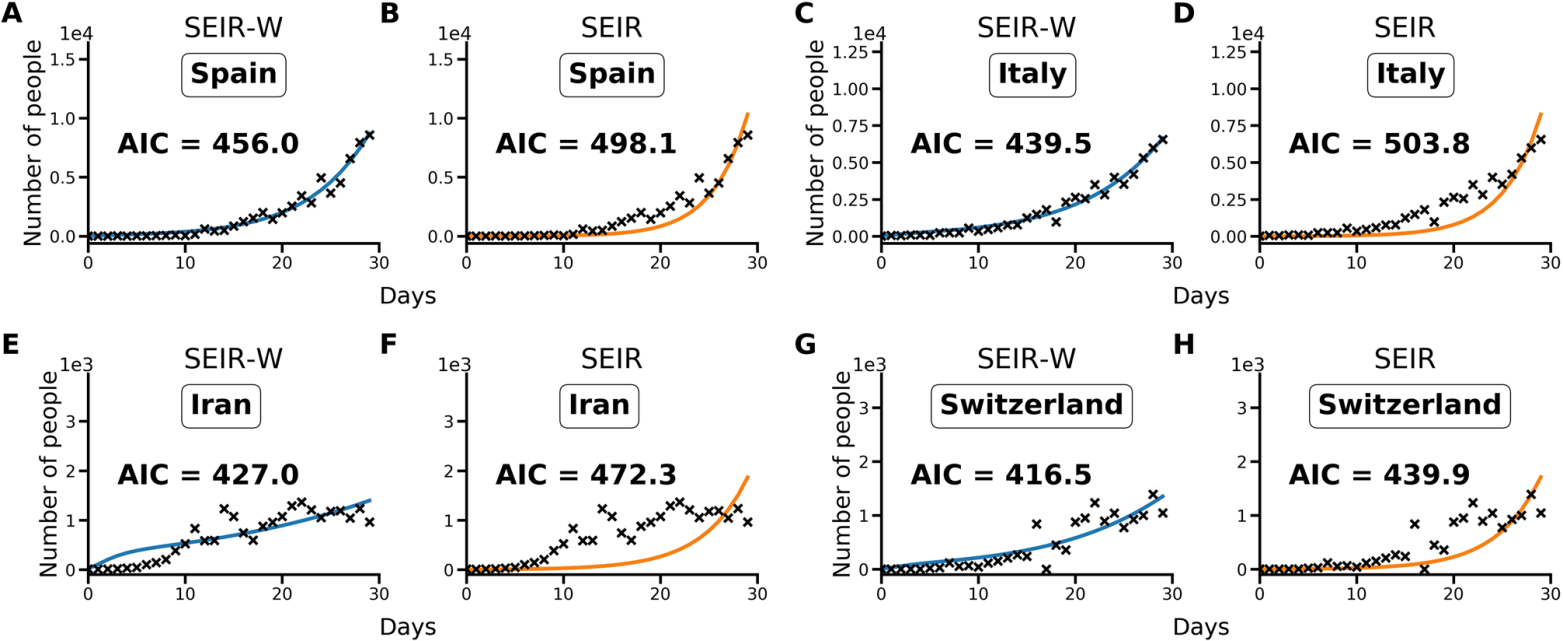
Illustrative model fit comparisons for SEIR-W and standard SEIR to case counts in early windows of the outbreak. The model fits are comparable across four countries with the largest early epidemics. These were chosen based having the highest cumulative number of infected cases after 30 days, following the first day when case counts were greater than or equal to 10. The four countries are (**a**,**b**) Spain, (**c**,**d**) Italy, (**e**,**f**) Iran and (**g**,**h**) Switzerland. These constitute a subset of 17 countries that had the highest number of cumulative COVID-19 cases (of the 181 total countries affected) as of March 30, 2020. Data come from the European Centre for Disease Control and Prevention, and from ourworldindata.org^14,65^. See supplementary information for more details.

Using the Akaike information criterion (AIC), SEIR models with an environmental compartment (SEIR-W) provide a strong relative fit to country incidence data. As discussed in the Methods and supplementary information, we compared the performance of models with (SEIR-W) and without (SEIR) environmental transmission across multiple countries to assess the role of environmental transmission in different contexts. Using the fitted parameters provided in Tables 1–3, and S1–S3, we calculate AIC values for the two mechanistic models: the standard SEIR model and the SEIR-W model. Table S4 displays the summary of the AIC values for each model-type fit to the first 30 days after the first day with total counts ≥ 10. In 10/17 countries (including 9/11 European countries), the SEIR-W model provided a better fit to the country data. In Fig. 1, we display the comparative individual country fit results for 4 of the countries with the fastest 30-day case growth rates—Spain, Italy, Iran, and Switzerland. The SEIR-W variant provides a better fit (significantly lower AIC score) than the standard SEIR model for all of these. Results for additional country fits can be found in the supplementary information, Fig. S2.

### Sensitivity analysis reveals how environmental transmission can modulate disease dynamics

**Figure 2:**
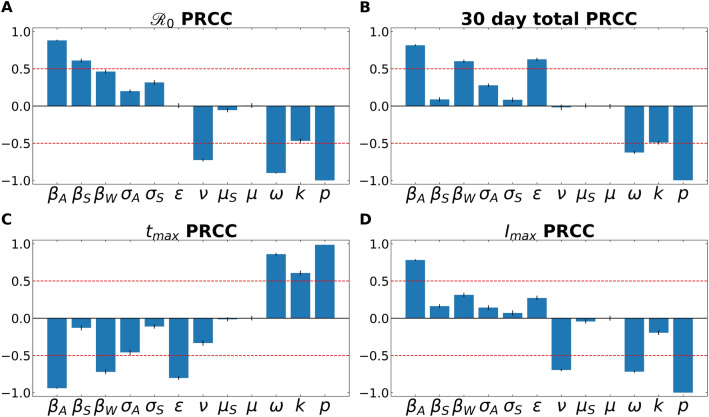
A partial rank correlation coefficient (PRCC) sensitivity analysis. PRCC was performed with respect to (**A**) ${\mathcal{R}}_{0}$, (**B**) total number of infected (and symptomatic) after 30 days of outbreak, (**C**) time to peak number of symptomatic individuals (*t*_*max*_), and (**D**) peak number of symptomatic individuals. This analysis highlights the intercorrelated sensitivities of each of the model parameters. The blue bars show the mean value of each PRCC, with error bars at one standard deviation. This analysis was performed by sampling over uniform distributions of 4.5% around the nominal model parameter values. Parameters correspond to the fixed ones in Table 3, and the average fitted parameters values in Table S5. The red line marks PRCC values of + /− 0.50 and helps identify parameters that are more influential (greater than 0.50 or less than—0.50). See supplementary information for more details.

Partial Rank Correlation Coefficient (PRCC) analyses for the four examined features of the outbreak—(1) ${\mathcal{R}}_{0}$, (2) total number of infected individuals after 30 days, (3) time to peak number of infected individuals, and (3) size of peak number of infected individuals. Figure 2 demonstrates the PRCC calculations for all four of these outbreak characteristics. For ${\mathcal{R}}_{0}$, we observe that the model was strongly sensitive to several aspects related to virus transmission—*β*_*A*_*, β*_*S*_*, β*_*w*_—as well as the rate at which asymptomatic individuals develop symptoms (⍵), the rate of recovery (4) and SARS-CoV-2 free-living survival rate (*k*).

One can also observe how some parameters are better suited to modify the peak of the infection, such as the recovery rate (ν; which includes in it the swiftness of diagnosing and treating the virus). Others modulate the timing of the peak, such as *ε*, the rate of leaving the “exposed” compartment (or equally well, the reciprocal of the average time spent in the exposed compartment). Note that across all features, the fraction of cases that move along the “mild” route (*p*), from *E → I*_*A*_* → R*, has a powerful influence on all factors.

### The ${\mathcal{R}}_{0}$ comprises person-to-person and indirect/environmental transmission

**Figure 3:**
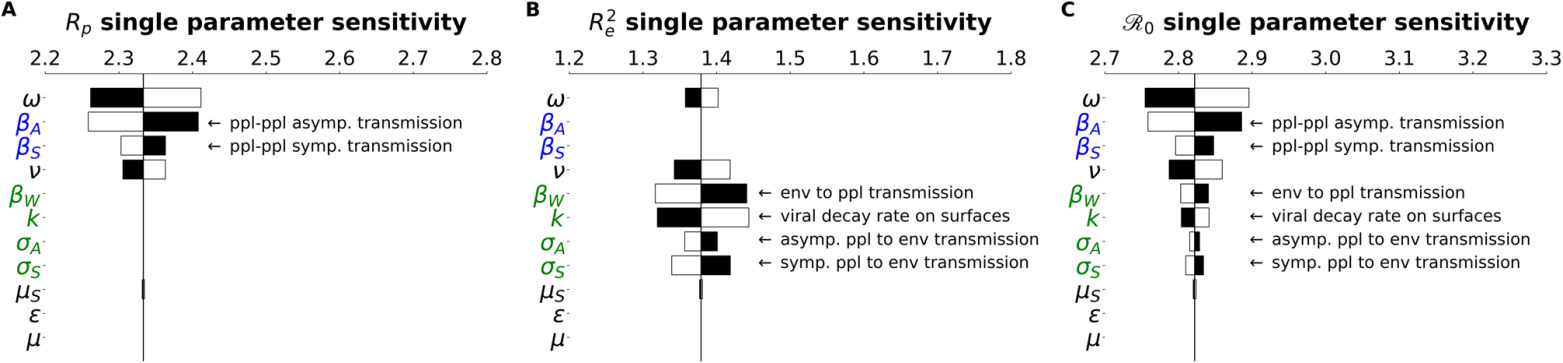
${\mathcal{R}}_{0}$ subcomponents have different parameter architecture. We compare the parameter architecture for the two ${\mathcal{R}}_{0}$ components that compose the full ${\mathcal{R}}_{0}$ expression, (**a**) *R*_*p*_, (**b**) *R*_*e*_^*2*^ and (**c**) ${\mathcal{R}}_{0}$_*.*_ Parameters are colored according to their relation with the environment or people: green parameters refer to the environment, blue parameters strictly refer to people, and black parameters are neutral in this regard. Black bars show the extent to which the component after changed when the parameter values are *increased* by 4.5%, The white bars show the same except for a *decrease* of 4.5%. For clarity, the single parameter that most influences the ${\mathcal{R}}_{0}$ and its subcomponents is the faction of cases that move through the mild route (*p*) has been removed. For more details on how this parameter influences the ${\mathcal{R}}_{0}$ and other features of the outbreak, see the PRCC analysis as discussed in the Methods and supplementary information.

In the Methods, we described how the ${\mathcal{R}}_{0}$ is composed of two sub-${\mathcal{R}}_{0}$ components, corresponding to different infectious interactions: person to person (*R*_*p*_), person to environment and environment to person (*R*_*e*_). Tornado plots were constructed that demonstrate how the ${\mathcal{R}}_{0}$-components have their own architecture and sensitivity (Fig. 3). These results highlight that *R*_*e*_ is sensitive to parameters associated with environmental transmission (*β*_*W*_*, *σ_*A*_*, *σ_*s*_*, k*). while *R*_*p*_ is sensitive to terms associated with direct transmission (⍵, *β*_*A*_, *β*_*S*_).

**Figure 4:**
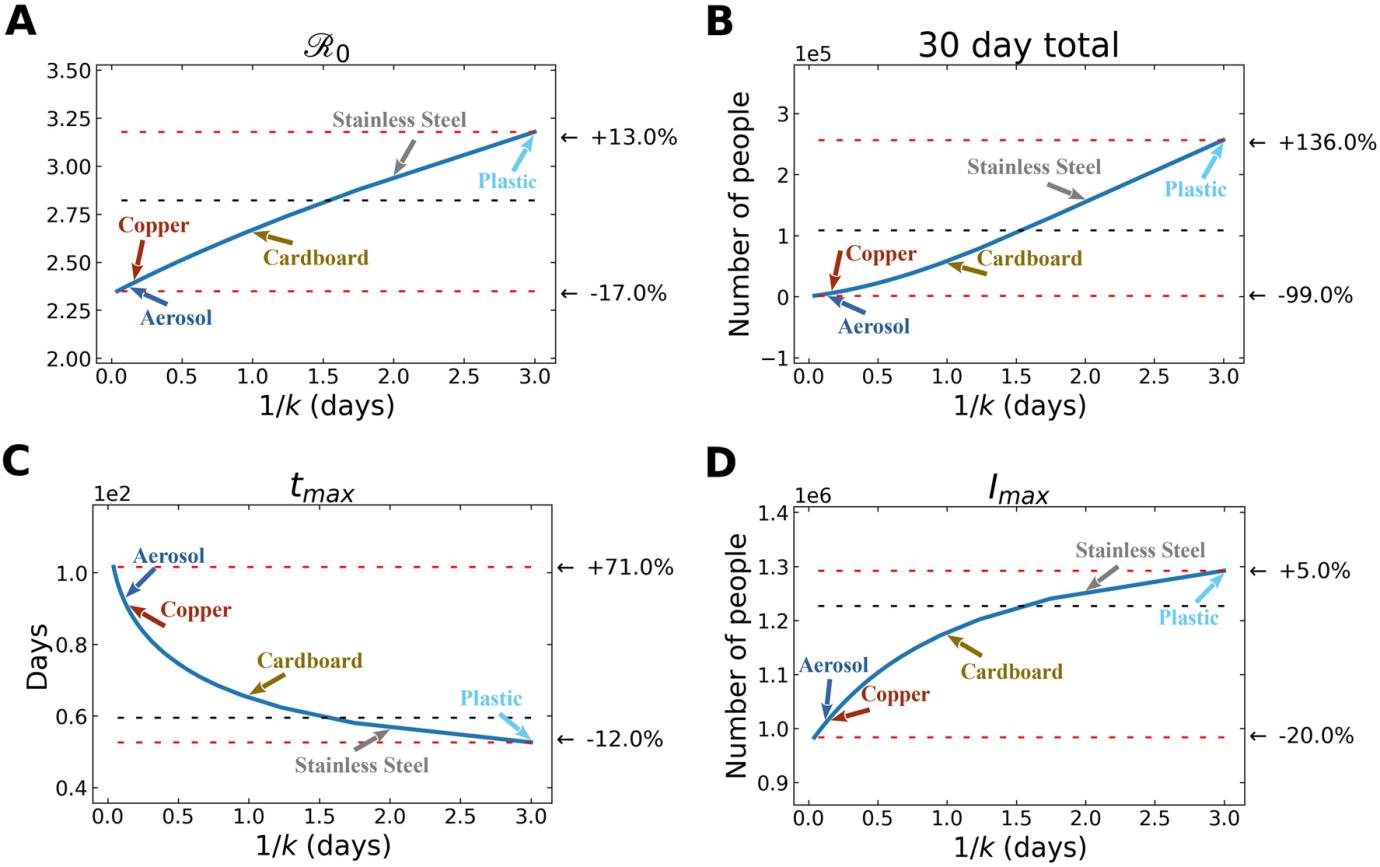
Environmental transmission: Various features of an outbreak change as a function of 1/*k* (where *k* is the rate of decay of SARS-CoV-2 survival in the environmental compartment): to (**A**) ${\mathcal{R}}_{0}$, (**B**) total number of infected (and symptomatic) after 30 days of outbreak, (**C**) time to peak number of symptomatic individuals (*t*_*max*_), and (**D**) peak number of symptomatic individuals. The black dashed lines show the value of the respective plotted value at the average value of 1/*k* (~ 1.5 days), used in the fits from above. The top red line shows the maximum of plotted value for either the smallest value of 1/*k* chosen (= *1 h)* or the largest value of 1/*k* chosen (= *3 days)*, depending on whether the plotted value decreases or increases with 1/*k*, and the bottom red line shows the plotted value at the other extreme of 1/*k*. We emphasize that these correspond to indirect, environmental transmission only. Aerosol transmission, for example, is likely a cause of direct transmission between individuals.

In Fig. 4, we observe how variation in free-living survival (1/*k*) influences four characteristics of an outbreak: ${\mathcal{R}}_{0}$, total number of infected individuals after 30 days, time to peak number of infected and symptomatic individuals, and maximum number of symptomatic individuals in the first 30 days. Note the annotations on the figure that highlight where the empirically determined survival times of SARS-CoV-2 on a range of reservoir types (aerosol, copper, plastic, cardboard, stainless steel)^30^. Also note that the quantitative relationships between 1/*k* and various outbreak features are slightly different. For example, the ${\mathcal{R}}_{0}$ increases more gradually across a wider range of free-living survival values than some of the other features (Fig. 4).

We should reemphasize some aspects of the underlying physics of the simulations in this study that were introduced in the Methods section. Studies have demonstrated that aerosol transmission is a key contributor to person-to-person transmission^52–56^. There are, however, scenarios where aerosols can serve as environmental reservoirs, capable of transmitting between individuals in an “indirect” way.

### The composition of abiotic reservoirs modulates outbreak dynamics

**Figure 5:**
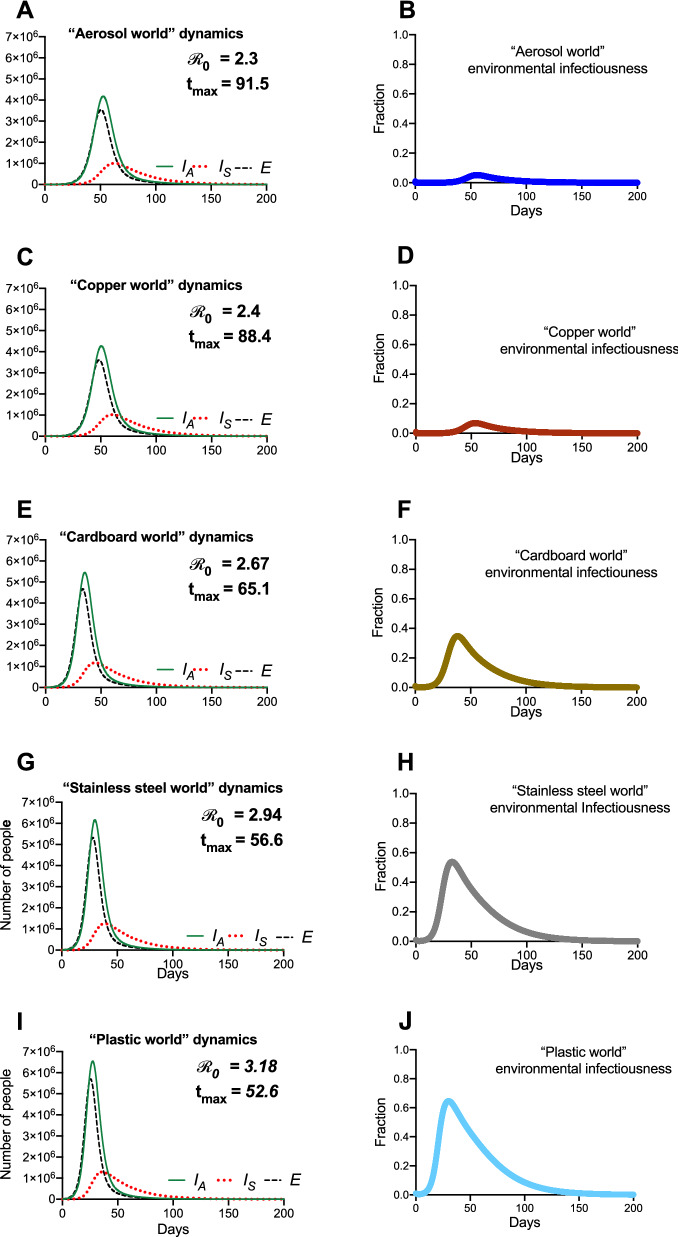
Hypothetical “reservoir world” simulations feature differing environmental transmission dynamics. Population and environmental dynamics of SEIR-W model outbreaks in hypothetical settings composed of pure substances where SARS-CoV-2 can survive and be transmitted. (**A**,**B**) “aerosol world,” (**C**,**D**) “copper world,” (**E**,**F**) “cardboard world,” (**G**,**H**) “stainless steel world,” and (**I**,**J**) “plastic world.” Environment infectiousness corresponds to the proportion of the environment that contains infectious SARS-CoV-2. Note that the surface where the viral decay is strongest (Copper), the peak of the epidemic is pushed farthest from the origin. Also note the ${\mathcal{R}}_{0}$ values graphs **A**, **C**, **E**, and **G**, which highlight that the different “reservoir worlds” behave like fundamentally different outbreaks in several ways.

Figure 5 depicts the results of “reservoir world” simulations, where the *k* values correspond to those from a 2020 study highlighting the survival of SARS-CoV-1 and SARS-CoV-2 on different physical surfaces^30^. While different physical surfaces might conceivably influence multiple parameters in our model, we focused only on the *k* values, as they are informed directly by available empirical data. In addition, modifying a single parameter allows for a simpler comparison of the dynamics between settings.

**Figure 6:**
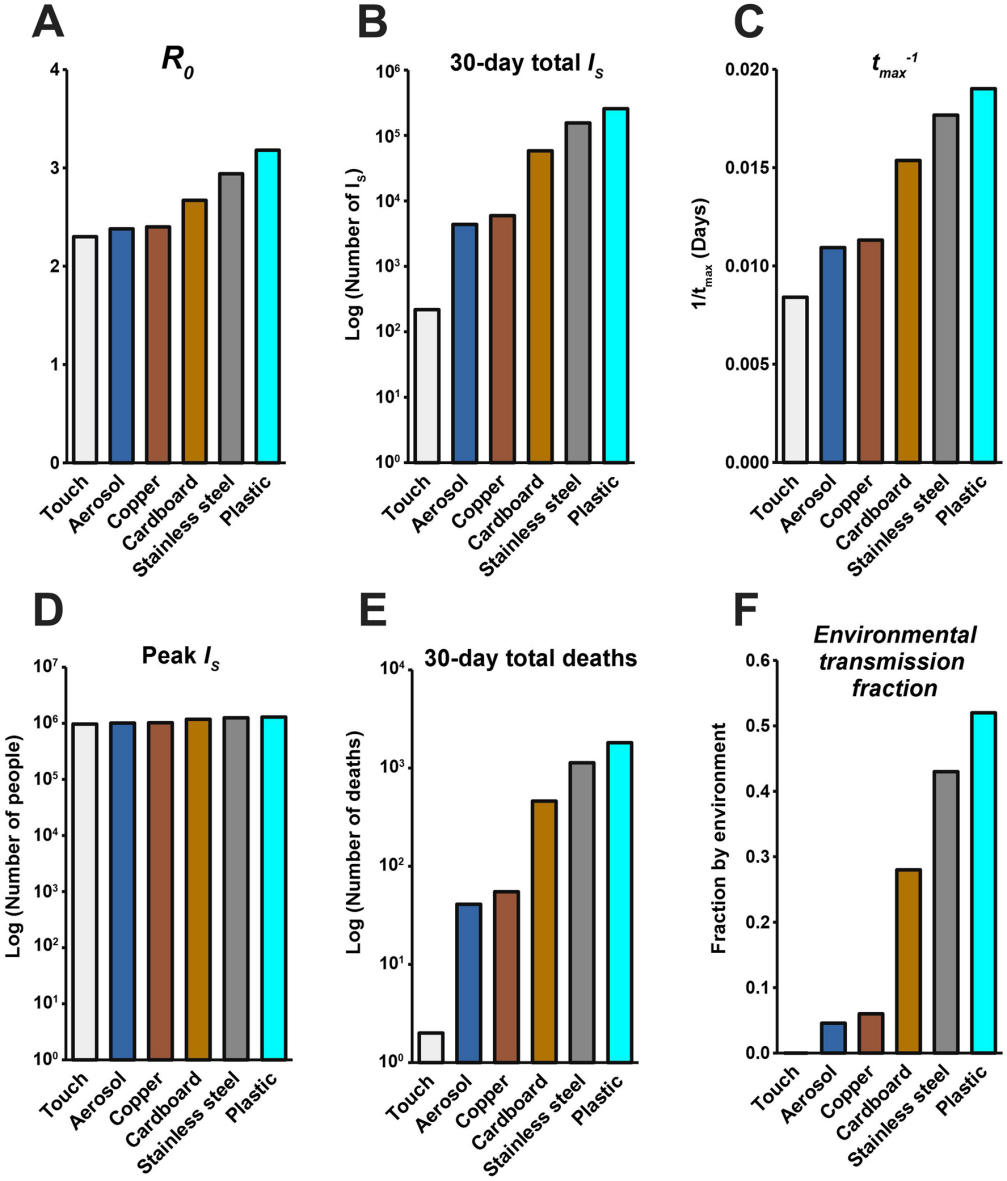
Summary of the hypothetical “reservoir world” outbreak intensity measures. *Graphs* correspond to the attributes of simulated epidemics where environments are entirely composed of a given physical surface, and larger values correspond to various aspects of outbreak intensity. (**A**) ${\mathcal{R}}_{0}$, (**B**) total number of infected (and symptomatic) after 30 days of outbreak, (**C**) the inverse time to peak number of symptomatic individuals (*t*_*max*_^*-1*^*;* larger values = shorter times to reach peak), (**D**) peak number of symptomatic individuals, (**E**) deaths after 30 days, and (**F**) environmental transmission fraction. Note the log scales on the y-axis in (**B**), (**D**) and (**E**).

The summary of these simulations (Fig. 5) highlights that the surface composition of a setting has a meaningful impact on several features of outbreak dynamics. Of the “reservoir world” simulations, “aerosol world” (5a) and “copper world” (Fig. 5c) takes the longest amount of time (91.5 and 88.4 days, respectively) to rise to the peak number of infected-symptomatic individuals, indicating an outbreak which is slower to develop. Relatedly, the ${\mathcal{R}}_{0}$ values are much different in the different “reservoir world” scenarios: The “aerosol world” simulation has an ${\mathcal{R}}_{0}$ of 2.38, the “copper world” simulation an ${\mathcal{R}}_{0}$ of 2.4, and the “plastic world” simulation an ${\mathcal{R}}_{0}$ of 3.18 (Fig. 6 and Table S5). In addition, the total number of individuals infected after 30 days of the outbreak, and the total number dead after 30 days are both significantly lower in the “aerosol world” and “copper world” setting (Fig. 6 and Table S5). The peak value of infected individuals is not dramatically different across “reservoir worlds.” That is, while many features associated with severity differ greatly across “reservoir world” settings, we observed lower variation in the peak of the epidemic as compared with the time to the peak of the epidemic (Table S5). Maybe the most noteworthy of the differences is the vast disparity in the number of deaths in the first 30 days of the outbreak, where the “plastic world” setting has more than 30 times the number of deaths as the “copper world” scenario (1814 vs. 55, respectively; Fig. 6 and Table S5).

Lastly, comparisons of the metric, *R*_*e*_^*2*^*/*(*R*_*e*_^*2*^ + *R*_*p*_), which measures the extent to which transmissions can be attributed to the environmental route, further highlights how different environmental reservoirs influence disease transmission (Fig. 6). The “aerosol world” and “copper world” settings comprise 4.6% and 6% of transmission events occurring through non-person-to-person transmission. This differs dramatically from the “plastic world” setting, where 52% of transmission events are occurring through the environmental route.

## Discussion

### Deconstructing the basic reproductive number (${\mathcal{R}}_{0}$) into subcomponents highlights the role of indirect transmission

By deconstructing the basic reproductive number into components, we can better understand how variation in the ${\mathcal{R}}_{0}$—by setting, time, or geography—may reside in how these contexts are driven by environmental transmission. Many of these effects may be (as they are in this study) localized to one component of the ${\mathcal{R}}_{0}$, labeled *R*_*e*_^*2*^ in this study. Notably, the *R*_*e*_^*2*^ component is highly sensitive to the transmission interaction between people and the environment (β_w_), and the decay rate of virus in the environment (*k*). Interestingly, the *R*_*e*_^*2*^ is relatively robust to the rate of infectious virus shed into the environment from the asymptomatic infected individuals (the parameter called *σ*_*A*_ in this model). Also, deconstructing the ${\mathcal{R}}_{0}$ value into these components facilitates the development of new metrics that quantify how much a given epidemic is driven by certain routes. As many viral diseases may contain multiple transmission routes, being able to properly quantify their relative contribution may be important for public health interventions. Adding dimensionality to the understanding of the ${\mathcal{R}}_{0}$ is a theme of modern epidemiology, as many recent findings have revealed the limitations of the standard definitions and sought to expand them^57,58^.

### Models that include indirect transmission recapitulate aspects of real-world outbreaks

In this study, we introduce a general framework for studying the joint effects of direct and indirect microparasite transmission and then analyzed that model with respect to real world SARS-CoV-2 outbreaks in different countries. We demonstrate that understanding the particulars of environmental transmission, including variation in viral free-living survival, can alter fundamental characteristics of disease dynamics. For SARS-CoV-2, we find that for 10 out of the 17 countries examined, the SEIR-W model better explained the epidemiological patterns than did SEIR models. Critically, both models include asymptomatic/sub-clinical transmission. Given that features of indirect transmission can influence central properties of disease dynamics, we should consider the possibility that variation in free-living survival may contribute to variation in the shape of real-world epidemics. Nevertheless, as features of individual country epidemics (e.g. quality of data, testing capacity) are myriad and difficult to disentangle, several aspects independent of the actual route of transmission could explain the superior fit of the SEIR-W models. The model is strongly sensitive to several aspects related to virus transmission, including the rate at which both symptomatic and asymptomatic individuals transmit infection to susceptible hosts, the rate at which asymptomatic individuals develop symptoms, the rate of recovery (ν) and SARS-CoV-2 decay rate (*k*). Even more, recent studies highlight how host behavior during a pandemic^59^ and the importance of urbanization^60^ can also influence disease dynamics. It is possible that the SEIR-W model captures various unknown features of epidemic dynamics, rooted in environmental transmission, or some other yet undiscovered mechanism.

### Disease dynamics in different hypothetical “reservoir world” settings resemble essentially different outbreaks

Questions about the existence and implications of indirect and aerosol transmission have been front-and-center in our attempts to understand COVID-19. Analysis of the ${\mathcal{R}}_{0}$ and its subcomponents highlights that many aspects of outbreak dynamics are sensitive to the parameter associated with environmental decay rate (*k* in the model presented in this study). Analysis of hypothetical settings purely comprising reservoirs of a certain kind (“reservoir world”) fortifies the significance of free-living survival on physical surfaces and environmental transmission in outbreak dynamics. While our findings cannot speak to the outbreak dynamics in any particular setting in the real world, they do reveal that the surface composition of a setting can significantly influence the behavior of a theoretical outbreak. For example, the ${\mathcal{R}}_{0}$ in the “plastic world” simulation (${\mathcal{R}}_{0}$ = 3.18) is over 1.3 times the ${\mathcal{R}}_{0}$ in the “copper world” simulation (${\mathcal{R}}_{0}$ = 2.4). Many other differences between these outbreaks come as a consequence of the different ${\mathcal{R}}_{0}$ values. The “plastic world” simulation reaches a peak number of symptomatic infectious individuals almost 1.7 times faster than the “copper world” simulation and kills over 30 times more people in the first 30 days (1814 deaths in “plastic world” vs. 55 deaths in the “plastic world”).

The differences between “reservoir world” epidemics are so significant that they might be naively interpreted as being caused by completely different pathogens. Note, however, that the maximum value of the infected- symptomatic populations are roughly equivalent across “reservoir worlds,” and so the influence of SARS-CoV-2 survival on physical surfaces (mediated by difference in free-living survival) doesn’t affect all aspects of outbreak dynamics equally.

Despite the breadth of differences observed across surfaces, the similarity between the “aerosol world” and “copper world” results is notable. That is, an outbreak in a hypothetical world where there are no physical surfaces, but only transmission via aerosols would be only slightly more intense than an outbreak where indirect transmission was driven entirely by copper (using ${\mathcal{R}}_{0}$ as a quick proxy, “aerosol world” = 2.38, and “copper world” = 2.4). The implications here are subtle, but worth elaborating on: while a lot of discussion has focused on a dichotomy between aerosol-mediated transmission and indirect transmission, our findings suggest that the differences between aerosol transmission and some physical surfaces is so minute that the epidemiological signature for differences between them may be indistinguishable. Consequently, conversations about transmission might be centered around a different point of contention: not on the existence of indirect transmission, but how the combination of aerosols and surfaces contribute to non-contact transmission events.

### Implications for the ecology of emerging outbreaks

Many months after COVID-19 has firmly established itself as a large and very costly pandemic, the scientific community remains in the fact-finding phase of SARS-CoV-2 biology and COVID-19 understanding. A significant source of fear and speculation in the pandemic involves the plausibility that SARS-CoV-2 has undergone local adaptation in certain settings, translating to different epidemiological properties. While there is no convincing molecular or clinical support for local adaptation in SARS-CoV-2, our findings highlight how easy it is to conflate an environmental (or ecological) difference for a genetic one: the same virus, spreading in populations of identical size and behavior, differing only in the composition of physical surfaces where the virus can be transmitted through the environment, can have ${\mathcal{R}}_{0}$ values between 2.4 and 3.18, with early death rates 30 times apart. Even further, our findings highlight how models of direct and indirect/environmental transmission can provide comparable results, rendering it easy to conflate routes of transmission^9,61,62^.

### Limitations and future directions

Though the findings provide insight into the dynamics and implications of environmental transmission in viral infections, this study—and others of its kind—have several limitations. For one, phenomenological models requiring the fitting of multiple parameters to limited (and noisy) data can be prone to spurious results. In addition, there are specific challenges involved with resolving the mechanism of transmission from simplistic models. The latter limitation is related to a challenge known as the identifiability problem that arises when models are fit to early outbreak data^9,61,62^.The difficulty in predicting outbreaks conferred by this identifiability problem, and the generic variability in disease dynamics from setting to setting has been encapsulated in a concept called permutation entropy^63^. Even more, environmental reservoirs composed of certain physical surfaces (plastic-like in our model) may be associated with phenomenon resembling a “superspreading” event, where individual variation in contagiousness can drive unusually large numbers of infections^64^. Perhaps a better understanding of how, and on what surfaces, viral populations survive may improve the predictability of outbreak trajectories.

Because of the identifiability problem^9,61,62^ and a general lack of consensus regarding the mechanisms of transmission in various diseases, we caution against the overextrapolation of these findings to any particular epidemic. As of fall 2020, the evidence for widespread surface transmission of SARS-CoV-2 remains dubious^30–41^. Insofar as SARS-CoV-2 is not the last of the emerging infectious diseases, then we should remain vigilant about understanding how different routes of transmission may influence disease dynamics, as there remain other microparasites in human and nonhuman hosts that are transmitted via the environmental route and require our attention.

## Supplementary information

Supplementary information.

## Data Availability

All data is available in the main text or the supplementary information. The code used for our analyses is publicly available on Github: https://github.com/OgPlexus/Copperland.
