# Supplementary material for: Variation in microparasite free-living survival and indirect transmission can modulate the intensity of emerging outbreaks
Source: Sci Rep. 2020 Nov 27;10:20786. doi: 10.1038/s41598-020-77048-4 (PMC7695845; doi:10.1038/s41598-020-77048-4)
Supplement: Supplementary file 1 — Supplementary information. [file 41598_2020_77048_MOESM1_ESM.docx]

Supplementary information

**Variation in microparasite free-living survival and indirect transmission can modulate the intensity of emerging outbreaks**

C. Brandon Ogbunugafor^1,2,3^*, Miles D. Miller-Dickson^2^, Victor A. Meszaros^2^, Lourdes M. Gomez^1,2^, Anarina L. Murillo^4,5^, and Samuel V. Scarpino^6^

^1^Department of Ecology and Evolutionary Biology, Yale University 06511

^2^Department of Ecology and Evolutionary Biology, Brown University 02912

^3^Center for Computational Molecular Biology, Brown University 02912

^4^Department of Pediatrics, Warren Alpert Medical School at Brown University 02912

^5^Center for Statistical Sciences, Brown University School of Public Health 02903

^6^Network Science Institute, Northeastern University 02115

^7^Roux Institute, Northeastern University 04101
^8^Santa Fe Institute 87501

Correspondence: brandon.ogbunu@yale.edu

**This file includes:**

- - Supplementary text
  - Fig. S1.SEIR-W model for SARS-CoV-2 transmission.
  - Fig. S2. Graphical depiction of data in the Table S4. A-Z correspond to 13 country fits of the mathematical models—SEIR and SEIR-W
  - Fig. S3. Histogram of the fractions of parameter space where AIC for the environmental model was less than the non-environmental model.
  - Table S1. Initial values used for susceptible and symptomatically infected individuals for each country.
  - Table S2. Fitted parameters used in the SEIR-W model for each of the 17 selected countries.
  - Table S3. Fitted parameters used in the model for each of the 17 selected countries. *β_W_*, 𝜎_A_, and 𝜎_S_ are set to zero when we run the standard SEIR country fits.
  - Table S4. The Akaike information criterion (AIC) for the fits conducted of the “early stage” (30 days) of the outbreak in each of the selected 17 countries with (SEIR-W) and without (SEIR) the WAIT component present. The larger of each pair of AIC scores is highlighted in red, and the smaller in green.
  - Table S5. Summary of the “reservoir world” simulations key features.
  - Supplementary references

**Supplementary Information**

*1. Author statement on mathematical modeling, and the purpose of this study.*
In any outbreak, mathematical modeling efforts are crucial for organizing the available information, transforming the unknowns into testable hypotheses, and providing projections of how the disease may progress under a set of assumptions [68, 69] This last point is an especially important, often under-treated aspect of computational and mathematical modeling of epidemics: predictions are useful, but only apply to sets of circumstances commensurate with the model assumptions.

Note that this study is not tasked with making any predictions about how any particular outbreak will happen in any setting. That models like the ones in this manuscript might apply to hypothetical scenarios doesn’t mean, however, that they are wholly irrelevant. We believe that our study offers a unique perspective on broader issues in the ecology of infectious disease that can be used to develop a better understanding of disease dynamics.

**Estimation of fixed parameters.**In the main text, we display the parameters and introduce the process through which parameters were estimated. Here we describe the process in better detail. There are 6 fixed parameters, 6 fitted parameters, and one parameter (⍵) dependent on the values of one of the fixed parameters (𝜂) and one of the fitted parameters (𝛆). These fixed parameters are 𝜂, 𝜇, 𝜇_S_, 𝜈, *k,* & *p*. The first, 𝜂, is the incubation period) [24, 67] and we assume that the expected time in the *E* state (1/𝛆) and the expected time in the *I_A_* state (1/⍵) sums to 𝜂, i.e. 𝜂 = 1/𝛆 + 1/⍵. Fixing 𝜂 constrains one of the two parameters, 𝛆 or ⍵, and the other can be fitted; we choose to fit 𝛆 and therefore constrain ⍵. The second fixed parameter 𝜇, the normal death rate, was calculated by taking the reciprocal of the average life expectancy (in days) of the 17 countries sampled, weighted by population size. We calculated a value of 80.3 years, based on data from individual countries [70]. The third parameter 𝜇_S_ is the sum of the normal death rate and an additional death rate due to a more severe form of the infection. Given the existence of this data on life expectancy, we felt strongly about including natural death dynamics, as they reflect the reality of the natural settings.

We assumed a death rate of 3.8% [67]. and that death follows after initial symptoms between 3 and 4 weeks [67]. Thus 𝜇_S_ = 𝜇 + 0.038/(3.5 * 7), where we use the average of 3 and 4 weeks and we convert to days with the factor of 7. The fourth fixed parameter 𝜈, the recovery rate once in the symptomatic state, was assumed to be the reciprocal of the average of 3 and 6 weeks (the range of recovery times) [22, 67] times the fraction of individuals in the symptomatic state that do not die, i.e. 1 - 0.038, so 𝜈 = (1 - 0.038)/(4.5 * 7). The fifth fixed parameter *k*, the rate of viral decay in the environment, is the reciprocal of the average time that SARS-CoV-2 is expected to survive in the environment across a set of abiotic reservoirs, based empirical measurements [46]. The sixth fixed parameter *p*, the fraction of individuals in the *I_A_* state that move on to recovery without experiencing severe symptoms, was taken to be 0.956 [20].

We fit our model variations to the daily new cases data provided (See: supplementary information) starting on the day when there were ≥10 cumulative infected cases in that region. We choose the starting point of 10 cumulative cases in order to allow the outbreak to settle into a more consistent doubling time while also providing enough of an early-on window to capture the dynamics relevant to the *ℛ_0_* and force of infection estimations.

We calculate the number of daily new infections in our model by numerically integrating the influx rate of new symptomatic infections over the course of a single day (i.e. ∫ *(1 - p) ⍵ I_A_* *dt*). We perform this calculation for each of 30 consecutive days and fit these values to the daily new cases data. We use the influx rate of symptomatic infections, as opposed to the total rate of new infection (including asymptomatic individuals), as we expect that the large majority of reported cases in the early COVID-19 outbreak to be symptomatic. And we expect that—in the outbreaks—almost all asymptomatic cases go unreported [44].

**Initial conditions.** For each country, we use the first cumulative count that is ≥10 as a proxy for the initial number of *active* symptomatic cases *I_S0_*. We can justify this by proposing that, given that the doubling time is expected to fall between 3 and 6 days [71] then the exponential growth rate parameter of the infection (*r in exp(rt)*) would fall between 0.231 days^-1^ and 0.116 days^-1^ respectively. And, assuming 1 initial infected individual, the time to reach 10 cases for the former rate would be about 10.0 days (~log(10)/0.231) and the time for the latter would be 19.8 days (~log(10)/0.116). Thus, since recovery of symptomatic individuals, which takes between 3 and 6 weeks [50, 67] exceeds this interval, we expect that at the point when 10 cases have accumulated, all cases are still active. Lastly, in fitting the data to our model, we initialize all fitting parameters to a value of 1.5, in whatever units are appropriate for that parameter (expected to be close to the true value for most of the fitting parameters).

As an estimate for the initial number active *asymptomatic* cases, we take *I_A0_ = I_S0_*. That is, we expect that there are approximately as many asymptomatic cases as symptomatic cases early on. This assumption appears to be consistent with empirical findings. For example, data from the Diamond Princess cruise liner [29, 72], where all passengers were tested, revealed that approximately half of positively-testing cases were asymptomatic. Lastly, we assumed that the initial number of exposed individuals was approximately *ℛ_0_· (I_A0_ + I_S0_)*, based on the supposition that each of the initially infectious individuals *(I_A0_ + I_S0_)* will have exposed the infection to approximately *ℛ_0_* other individuals. We take the value of *ℛ_0_* in this case to be 2.5, based on prior studies [42, 44]. The *R* population is assumed to be 0 in the early stage of the outbreak, given the (average) 3 to 6-week recovery delay of COVID-19. The *W* population is assumed to be 1%.

Note: For clarity, we will add material that also appears in the main text below. This will facilitate an easier reading of the mathematical details as outlined here in thes supplemental material.

In our model, the indirect infection via aerosols are encoded into the terms associated with the *W* component, just as the different physical surfaces are. Alternatively, aerosol transmission that leads to direct infection between individuals is encoded in the terms associated with direct infection between susceptible individuals and those infected (see section entitled **Infection Trajectories** in the main text).

Environmental reservoirs infect people through the *β_W_* term (equations 1 and 2), a proxy for a standard transmission coefficient, corresponding specifically to the probability of successful infectious transmission from the environment reservoir to a susceptible individual (the full rate term being *β_W_W·S*). Hence, the *β_W_* factor is defined as the fraction of people who interact with the environment daily, per fraction of the environment, times the probability of transmitting infection from environmental reservoir to people. The factor *β_W_W* (where *W* is the fraction of environmental reservoirs infected) represents the daily fraction of people that will interact with the infected portion of the environment and become infected themselves. The full term *β_W_W·S* is thus the total number of infections caused by the environment per day.

In an analogous manner, we model the spread of infection *to* the environment with the two terms 𝜎*_A_ I_A_·(1 - W) / N* and 𝜎*_S_ I_S_·(1 - W) / N* representing deposition of infection to the environment by asymptomatic individuals, in the former, and symptomatic individuals, in the latter. In this case, 𝜎*_A_* (and analogously for 𝜎_S_) gives the fraction of surfaces/reservoirs that interact with people at least once per day, times the probability that a person (depending on whether they are in the *I_A_* or the *I_S_* compartment) will deposit an infectious viral load to the reservoir. Thus, 𝜎*_A_ I_A_ / N* and 𝜎*_S_ I_S_ / N* (where *N* is the total population of people) represent the daily fraction of the environment that interacts with asymptomatic and symptomatic individuals, respectively. Lastly, the additional factor of *(1 - W)* gives the fraction of reservoirs in the environment that have the potential for becoming infected, and so 𝜎*_A_ I_A_·(1 - W) / N* (and analogously for *I_S_*) gives the fraction of the environment that becomes infected by people each day. We use *W* to represent a fraction of the environment, although one could also have multiplied the *W* equation by a value representing the total number of reservoirs in the environment (expected to remain constant throughout the course of the epidemic, assuming no intervention strategies).

*1. Elaborated derivation of formulas 8a and 8b (from the main text).*

Here we provide a detailed derivation of several equations described in the main text. First, we provide equations 8a and 8b, as described in the main text.:

$R_{p}=\frac{\epsilon\left( \beta_{A}\left( \mu_{S}+\nu\right) + \beta_{S}\left( 1-p \right)\omega\right)}{\left( \mu+\epsilon\right)\left( \mu+\omega\right)\left( \mu_{S}+\nu\right)}$ , $R_{e}^{2}=\frac{\epsilon\beta_{W}\left( \sigma_{A}\left( \mu_{S}+\nu\right) + \sigma_{S}\left( 1-p \right)\omega\right)}{k\left( \mu+\epsilon\right)\left( \mu+\omega\right)\left( \mu_{S}+\nu\right)}$ (8a, 8b)

First, *R_p_* represents the number of secondary infections of people through person-to-person contact. This avenue is captured by the *β_A_* and *β_S_* terms in equations 1 and 2 in the main text. Namely, the rate of converting susceptible individuals to exposed individuals through person-to-person contact with infectious individuals is given by *β_A_ S I_A_ / N* for asymptomatic individuals and by *β_S_ S I_S_ / N* for symptomatic individuals. Near the disease-free-equilibrium (DFE), *N ~ S_0_* (i.e. the total population is initially susceptible) and so the rate of infection conversion for each, near the DFE, is given (approximately) by *β_A_* *I_A_* and by *β_S_ I_S_*. And so, the rate of conversion *per* infectious individual (*I_A_* and *I_S_*) is given simply by *β_A_* and by *β_S_* respectively. The average time that an individual remains in the infectious state is given by the reciprocal of the “exit”-rate of that state, which is 1/(*μ* + ⍵) for *I_A_*, and by 1/(*μ_S_* + 𝜈) for *I_S_*.

Thus the average number of secondary infections by individuals in the asymptomatic-infectious state is given by *β_A_ /(μ* + *⍵)* and by *β_S_/(μ_S_ + 𝜈)* for the symptomatic-infectious state. It is not sufficient to simply add these quantities together as the rates/probabilities of entering the *I_A_* and the *I_S_* differ from one another. In order to properly combine these two rates, we need to determine the fraction of individuals, early-on, that will be in each infectious compartment—or, if one prefers, the *probability* of being in either infectious state. Individuals entering the *I_A_* compartment can only do so by leaving the *E* compartment. And individuals can only leave the *E* compartment by entering the *I_A_* compartment or through death.

The fraction of individuals that move on to the *I_A_* compartment is therefore given by *ε / (μ + ε)*, i.e. the ratio of the rate of entering the *I_A_* compartment to the rate of leaving the *E* compartment, per person. And, the fraction of individuals that move on from the *I_A_* compartment to the *I_S_* compartment is, analogously, *(1 - p) ⍵ / (μ + ⍵)*, i.e. the ratio of the rate of entering the *I_S_* compartment to the rate of leaving the *I_A_* compartment. Note that individuals who make it to the *I_S_* compartment must also have made it through the *I_A_* compartment, and only a fraction *ε /* *(μ + ε)* do. So the rate of new infections caused by asymptomatic individuals and by symptomatic individuals needs to be weighted accordingly; *ε / (μ + ε)* for the former and *ε / (μ + ε) x (1 - p) ⍵ / (μ + ⍵)* for the latter—the latter needs both factors since individuals who make it to *I_S_* must also make it through *I_A_* first. Thus, weighting the rates in a sum accordingly, one finds that the rate of secondary person-to-person infections is given by, [ *β_A_ /(μ + ⍵) x ε / (μ + ε) ] + [ β_S_/(μ_S_ + 𝜈) x ε / (μ + ε) x (1 - p) ⍵ / (μ + ⍵)* ], the first term in brackets corresponding to infections by asymptomatic individuals, and the second term to symptomatic individuals. This expression simplifies to the form given in equation 8a.

A similar story can be told for the number of secondary infections of people, mediated by the environment, *R_e_^2^*. Although, in this case, we can break down this reproductive ratio further into two sub-components of its own: one representing the flow of infection *to* the environment *from* people, and another *from* the environment *to* people. We will call the former *R_pe_* and the latter *R_ep_*.

The derivation for *R_pe_* is identical to *R_p_* described above except that we replace *β_A_* and *β_S_* by *σ_A_/S_0_* and *σ_S_/S_0_* respectively, since these latter quantities represent the rates of depositing infection to the environment (near the DFE), as opposed to the rates of depositing infection to people as in the case of *β_A_* and *β_S_*. The derivation is otherwise identical since the probability of an individual making it to the *I_A_* or the *I_S_* compartments remains unchanged. *σ_A_/S_0_* and *σ_S_/S_0_* can be seen to represent the rate of environmental infection per infectious individual (for asymptomatic and symptomatic individuals respectively) by examining the full rate in equation 6 (in the main text), namely (*σ_A_ I_A_ + σ_S_ I_S_) (1 - W) /N.* Near the DFE, the factor *1 - W* is nearly equal to 1, and *N ~ S_0_*. Thus, as above, the individual rates of infecting the environment *per* infectious person is given by *σ_A_/S_0_* for asymptomatic individuals and by *σ_S_/S_0_* for symptomatic individuals. The apparent dependence on *S_0_* in the *ℛ_0_* is a curious feature of the model, although as we will soon see, this dependence cancels out entirely, leaving the full *ℛ_0_* devoid of any such dependence on population density. Following the lines of the derivation for *R_p_* described above, but with *β_A_* and *β_S_* replaced by *σ_A_/S_0_* and *σ_S_/S_0_* respectively, one arrives at,

$\frac{\epsilon(\sigma_{A}(\mu_{S}+\nu)+\sigma_{S} (1-p) \omega)}{S_{0}(\mu+\epsilon)(\mu+\omega)(\mu_{S}+\nu)}$(S1)

This quantity can be interpreted as the *fraction* of the environment infected by a single infectious individual near the DFE. Note that we do not specify their infectious-type (asymptomatic or not) as this feature is implicit in the weighted sum we took to arrive at equation S1 (and equation 8a), representing the *expectation value* of the fraction of new environmental infections. Note also that we are justified in discussing increments in the *fraction* of the environment infected as these calculations are performed near the DFE, where we can assume only a very small fraction of the environment is infectious and thus need not be concerned by scaling the fractional quantities to values exceeding unity.

Lastly, the derivation for *R_ep_* is straightforward. The rate of infection from the environment is given by *β_W_ W S* (equations 1 and 2 in the main text). Near the DFE, *S ~ S_0_*, and so the rate of people infected by the environment *per fraction* of the infected environment (*W*) is given simply by *β_W_ S_0_*. The average time any fraction of the infected environment remains infected is given by *1/k*, i.e. the reciprocal of the exit rate of the *W* compartment. Thus, the number of new infections of people, per fraction of the environment, near the DFE, is given simply by

$\frac{\beta_{W}S_{0}}{k}$ (S2)

Thus, the number of *people* infected by an individual who has first deposited infection to the environment (parameterized here as some “fraction” of the environment) is simply the product of the fraction of the environment infected per infectious individual (equation S1) and the number of people infected per infectious fraction of the environment (equation S2).

$\frac{\epsilon\beta_{W}\left( \sigma_{A}\left( \mu_{S}+\nu\right)+\sigma_{S}\left( 1-p \right)\omega\right)}{k\left( \mu+\epsilon\right)\left( \mu+\omega\right)\left( \mu_{S}+\nu\right)}$ (S3)

The dependency on *S_0_* cancels out in the product. A reproductive ratio representing the number of new infections of people per infectious person that are mediated through the environment is the product of two reproductive ratios: one representing the spread of infection from people to the environment, and another representing the spread of infection from the environment to people. If one follows the lines of previous efforts for calculating reproductive ratios in general ODE-systems [69], then they would find that for the system of ODEs (equations 1-6 in the main text), the full *ℛ_0_* expression is composed of the two subcomponents (*R_p_* and *R_e_^2^*) described here in a way expressed by equation 7 in the main text.

A brief explanation of the final *ℛ_0_* form, which is made clearer by following methods as described in prior studies [73], is that it is the maximum eigenvalue of the next-generation matrix *G*:

$G=\left( R_{p} R_{ep} R_{pe} 0 \right)$ (S4)

This matrix represents the amount by which the infected populations (taken as a 2 x 1 vector of inputs to the matrix) are scaled from one infection generation to the next [69]. For simplicity, here we only consider two infectious components: people and the infectious portion of the environment, as opposed to splitting people into all of the infected categories in our model (*E, I_A_, & I_S_*), simplifying *G* to a 2 x 2 matrix. As explained in more detail in Diekmann et al. 2010 [73] this simplification will preserve the maximum eigenvalue of the system, although one could just as well follow the lines of the calculation using the full 4 x 4 version of *G*, accounting for all infected compartments *E, I_A_, I_S_, & W*.

One will notice that *G* in equation S4 includes the three subcomponents of the *ℛ_0_* discussed above (equation 8a, and equations S1 & S2). Each element represents an *ℛ_0_* from *a* to *b* where *a* and *b* could represent people or the environment, making the 0-0 component of *G* (using indices beginning at 0) the *ℛ_0_* of people to people, the 0-1 component the *ℛ_0_* of the environment to people, the 1-0 component the *ℛ_0_* of people to the environment, and lastly the 1-1 component gives the *ℛ_0_* of the environment to the environment, which is zero in this case as the environment does not infect itself. Note that under this basis, the vector of inputs is the column vector given by (infected people at time t, infected fraction of environment at time t). As is readily verifiable, solving for the maximum eigenvalue of *G* gives equation 7 in the main text, with the identification that *R_e_^2^* = *R_ep_R_pe_*.

*2. Model fitting and parameter estimation*

Mathematical models like the ones developed in this study require the use of parameters, terms that dictate the way that the different parts of the model interact. In the case of SARS-CoV-2 transmission, we are fortunate that many early studies have provided estimates for many terms, like the incubation period, and rate of recovery. That said, there remains many terms for which there are no solid estimates. In this scenario, we must estimate these values. There are many ways to attempt this. One way to do this is to fit the model (using the fixed parameters based on values that we do have less uncertainty for) in order to estimate the unknown parameters. To do this, we use real-world data on the COVID-19 outbreak from 17 countries, in their “early-stage” outbreaks.

We define the “early stage” of the epidemic as the 30 days following the first day with ≥10 cumulative infected individuals within a particular region. This allowed us to standardize our comparisons between different regions, leading to more robust fitting results. We choose case counts ≥10 in order to avoid early difficulties with testing and recording and to give the infection sufficient time to settle into a more consistent doubling time. The window of 30 days was chosen in order to maximize the number of data points while also allowing enough room to include countries who have had a long enough exposure to SARS-CoV-2 to be included in the analysis.

We conduct our analysis using early stage data (30 days) from the following 17 countries (in alphabetical order): Australia, Austria, Canada, China, Denmark, France, Germany, Iran, Italy, Netherlands, Norway, Spain, Sweden, South Korea, Switzerland, the United Kingdom, and the United States. These countries were chosen because they had both the highest cumulative COVID-19 cases (of the 181 total countries affected) as of 03/30/2020, and because the outbreak had developed for at least 30 days following the first day with ≥10 cumulative infected cases within each country [14, 65].

Fitting our model to data from each of these countries, we deduce values for 6 model parameters, *β_A_, β_S_, β_W_, σ_A_, σ_S_, and* 𝜀 in the SEIR-W case, and 3 model parameters *β_A_, β_S_, and* 𝜀 in the SEIR case. Using the fitted parameters, we calculate Akaike Information Criterion (AIC) values (equation S6, discussed below) for the two versions of the model: one with the environmental reservoir included (SEIR-W) and one without it (SEIR). The AIC is an estimator representing the quality of a statistical model given a particular set of data and thus provides a means for model selection. Note that the AIC takes into account the number of fitting parameters when assessing how effective the model captures the information of the data. Consequently, it allows one to responsibly compare the fits for the two models (SEIR and SEIR-W) even though they contain different numbers of parameters.

The environment can be “turned off” by setting certain parameters (arrows in the compartmental diagram) to 0. These include *β_W_,* 𝜎_A_, and 𝜎_S_; each representing some coupling between the environment and people. The reciprocal of 𝜀 gives the number of days an individual is expected to remain in the *E* compartment before becoming asymptomatically infectious. We include this parameter in both fits (SEIR-W and SEIR) since little is known about how long the period is, after being initially exposed to SARS-CoV-2, before an individual becomes infectious (which we assume occurs *before* symptom onset). The only constraint on 𝜀 in the curve fitting is that 1/𝜀 lies between 0 and 5.5 days (the incubation period). We set 1/ω (the expected time in the *I_A_* compartment) to be the remainder of the time in the incubation period; i.e. *ω^-1^ = η - 𝜀^-1^* where η is the incubation period (5.5 days). All other fitting parameters are constrained to lie between 0 and 100, which is expected to provide ample room in the parameter space to locate an appropriate fit.

We use the python module *scipy.optimize.curve fit* to fit our model to the data from each country. This program uses the Levenberg-Marquardt algorithm to perform a least-squares regression analysis [74]. Using the optimal parameters, we computed the log-likelihood, *L*, using the following formula:

$L=-\frac{N}{2}lnln 2\pi-Nlnln \sigma-\frac{\sum_{t=1}^{N} \left( y_{t}-f\left( t,q \right) \right)^{2}}{2\sigma^{2}}$ (S5)

$f\left( t,q \right)$ is the number of people infected on day *t* predicted by the model, *y* represents the data, *N* is the number of data points (in this case, 30), and σ^2^ is taken to be the maximum-likelihood estimation (MLE) of the variance, given by [75].

$\sigma_{MLE}^{2}=\frac{1}{N}\sum_{t=1}^{N} \left( y_{t}-f\left( t,q \right) \right)^{2}$(S6)

From this we compute an AIC value using the formula,

$AIC=2(k_{\theta}-L)$ (S7)

where *k*_𝞱_ is the number of fitting parameters (6 for SEIR-W and 3 for SEIR) and *L* is the log-likelihood with MLE-calculated variance shown above. As mentioned above, we compute AIC values with and without the environmental reservoir. In each case, we perform the least-squares regression and arrive at the optimal parameters: *β_A_, β_S_, β_W_, σ_A_, σ_S_*, and 𝜀 in the former, and *β_A_, β_S_*, and 𝜀 in the latter.

Having established parameter values from this analysis, our primary aim is to assess the impact of SARS-CoV-2 environmental transmission (via copper, steel, cardboard, and plastic) on both the general (averaged) and country specific dynamics of the COVID-19 pandemic.

As stated previously, these 17 countries were chosen because they both had the highest cumulative COVID-19 cases (of the 181 total countries affected) as of the end of March, 2020.

| **Country** | **Value of S_0_ (people)** | **Value of I_S0_** | **Source (S_0_** & **I_S0_)** |
| --- | --- | --- | --- |
| Australia | 25,499,881 | 11 | [14] |
| Austria | 9,006,400 | 10 | [14] |
| Canada | 37,742,157 | 11 | [14] |
| China | 60,000,000 | 27 | [14] |
| Denmark | 5,792,203 | 10 | [14] |
| France | 65,273,512 | 11 | [14] |
| Germany | 83,783,945 | 11 | [14] |
| Iran | 83,992,953 | 18 | [14] |
| Italy | 60,461,828 | 17 | [14] |
| Netherlands | 17,134,873 | 13 | [14] |
| Norway | 5,421,242 | 15 | [14] |
| South Korea | 51,269,183 | 12 | [14] |
| Spain | 46,754,783 | 12 | [14] |
| Sweden | 10,099,270 | 12 | [14] |
| Switzerland | 8,654,618 | 12 | [14] |
| United Kingdom | 67,886,004 | 13 | [14] |
| United States | 331,002,647 | 11 | [14] |

**Table S1**. Initial values used for susceptible and symptomatically infected individuals for each country.

| **Country** | *β_A_* | *β_S_* | *β_W_* | 𝜎_A_ | 𝜎_S_ | *1/ε* | **Source** |
| --- | --- | --- | --- | --- | --- | --- | --- |
| Australia | 0.233 | 1.231 | 0.000 | 2.143 | 33.960 | 5.445 | [Fitted] |
| Austria | 0.000 | 0.000 | 0.071 | 2.983 | 0.001 | 0.000 | [Fitted] |
| Canada | 1.503 | 5.015 | 0.001 | 0.359 | 30.454 | 5.032 | [Fitted] |
| China | 0.915 | 0.000 | 0.000 | 1.641 | 12.829 | 2.403 | [Fitted] |
| Denmark | 0.211 | 0.000 | 0.128 | 0.000 | 11.471 | 2.749 | [Fitted] |
| France | 0.777 | 0.000 | 0.000 | 0.079 | 26.404 | 2.426 | [Fitted] |
| Germany | 0.649 | 0.000 | 0.000 | 4.349 | 75.298 | 2.451 | [Fitted] |
| Iran | 0.470 | 0.000 | 0.036 | 0.000 | 0.000 | 2.749 | [Fitted] |
| Italy | 0.479 | 0.000 | 0.032 | 0.000 | 0.482 | 1.744 | [Fitted] |
| Netherlands | 0.000 | 0.000 | 0.037 | 6.054 | 0.001 | 0.003 | [Fitted] |
| Norway | 0.525 | 0.000 | 0.075 | 0.000 | 0.000 | 2.748 | [Fitted] |
| South Korea | 0.752 | 2.100 | 0.000 | 2.306 | 3.804 | 2.434 | [Fitted] |
| Spain | 0.724 | 0.000 | 0.020 | 0.000 | 2.243 | 2.591 | [Fitted] |
| Sweden | 0.523 | 0.000 | 0.027 | 0.000 | 9.322 | 2.747 | [Fitted] |
| Switzerland | 0.590 | 0.000 | 0.100 | 0.000 | 0.000 | 2.747 | [Fitted] |
| United Kingdom | 0.421 | 0.000 | 0.002 | 27.754 | 8.622 | 1.323 | [Fitted] |
| United States | 0.573 | 0.000 | 0.000 | 10.199 | 14.474 | 2.540 | [Fitted] |

**Table S2. Fitted parameters used in the SEIR-W model for each of the 17 selected countries.**

| **Country** | *β_A_* | *β_S_* | *β_W_* | 𝜎_A_ | 𝜎_S_ | *1/ε* | **Source** |
| --- | --- | --- | --- | --- | --- | --- | --- |
| Australia | 3.042 | 1.058 | 0.000 | 0.000 | 0.000 | 5.445 | [Fitted] |
| Austria | 0.000 | 12.203 | 0.000 | 0.000 | 0.000 | 0.009 | [Fitted] |
| Canada | 0.000 | 9.176 | 0.000 | 0.000 | 0.000 | 5.445 | [Fitted] |
| China | 0.915 | 0.000 | 0.000 | 0.000 | 0.000 | 2.404 | [Fitted] |
| Denmark | 0.000 | 9.626 | 0.000 | 0.000 | 0.000 | 0.022 | [Fitted] |
| France | 0.777 | 0.000 | 0.000 | 0.000 | 0.000 | 2.426 | [Fitted] |
| Germany | 0.649 | 0.000 | 0.000 | 0.000 | 0.000 | 2.451 | [Fitted] |
| Iran | 0.000 | 12.323 | 0.000 | 0.000 | 0.000 | 5.307 | [Fitted] |
| Italy | 0.233 | 16.127 | 0.000 | 0.000 | 0.000 | 5.445 | [Fitted] |
| Netherlands | 0.001 | 12.427 | 0.000 | 0.000 | 0.000 | 0.008 | [Fitted] |
| Norway | 0.001 | 9.250 | 0.000 | 0.000 | 0.000 | 0.009 | [Fitted] |
| South Korea | 1.097 | 1.956 | 0.000 | 0.000 | 0.000 | 3.389 | [Fitted] |
| Spain | 0.000 | 17.840 | 0.000 | 0.000 | 0.000 | 0.014 | [Fitted] |
| Sweden | 0.000 | 9.680 | 0.000 | 0.000 | 0.000 | 0.106 | [Fitted] |
| Switzerland | 0.000 | 12.949 | 0.000 | 0.000 | 0.000 | 0.014 | [Fitted] |
| United Kingdom | 0.000 | 11.715 | 0.000 | 0.000 | 0.000 | 5.445 | [Fitted] |
| United States | 0.574 | 0.000 | 0.000 | 0.000 | 0.000 | 2.544 | [Fitted] |

**Table S3. Fitted parameter values, SEIR (by country).** Fitted parameters used in the model for each of the 17 selected countries. *β_W_*, 𝜎_A_, and 𝜎_S_ are set to zero when we run the standard SEIR country fits.

*3. Model fitting and parameter estimation II*

Tables S1-S3 display all the data relevant to the model initial conditions (for each country), and the AIC values for the country fits. In Fig. S3, we show graphs corresponding to individual country fits. Note that four countries with the most explosive early outbreaks—Spain, Italy, Iran and Switzerland—appear in the main text (Fig. 1).

Explosiveness was defined by the highest cumulative number of infected cases after 30 days following the first day when cases were greater than or equal to 10). These four also appear in the table, however, so that their fits can be compared to the other 14 countries in the set.

|  | **Australia** | **Austria** | **Canada** | **China** | **Denmark** |
| --- | --- | --- | --- | --- | --- |
| AIC SEIR-*W* | 94.6 | 400 | 311.9 | 388.5 | 332.6 |
| AIC SEIR | 88.5 | 422 | 307 | 382.5 | 349.9 |

|  | **France** | **Germany** | **Iran** | **Italy** | **Netherlands** |
| --- | --- | --- | --- | --- | --- |
| AIC SEIR-*W* | 273.7 | 221.4 | 427 | 439.5 | 377.9 |
| AIC SEIR | 267.7 | 215.4 | 472.3 | 503.8 | 420.8 |

|  | **Norway** | **South Korea** | **Spain** | **Sweden** | **Switzerland** |
| --- | --- | --- | --- | --- | --- |
| AIC SEIR-*W* | 344.1 | 356.1 | 456 | 322.7 | 416.5 |
| AIC SEIR | 363.4 | 350.1 | 498.1 | 339 | 439.9 |

|  | **United Kingdom** | **United States** |
| --- | --- | --- |
| AIC SEIR-*W* | 372.8 | 191.9 |
| AIC SEIR | 375 | 185.9 |

**Table S4. The Akaike information criterion (AIC) for the country data fits.** The AIC fits were conducted on the “early stage” (30 days) of the outbreak in each of the selected 17 countries with (SEIR-W) and without (SEIR) the WAIT component present. The larger of each pair of AIC scores is highlighted in red, and the smaller in green.


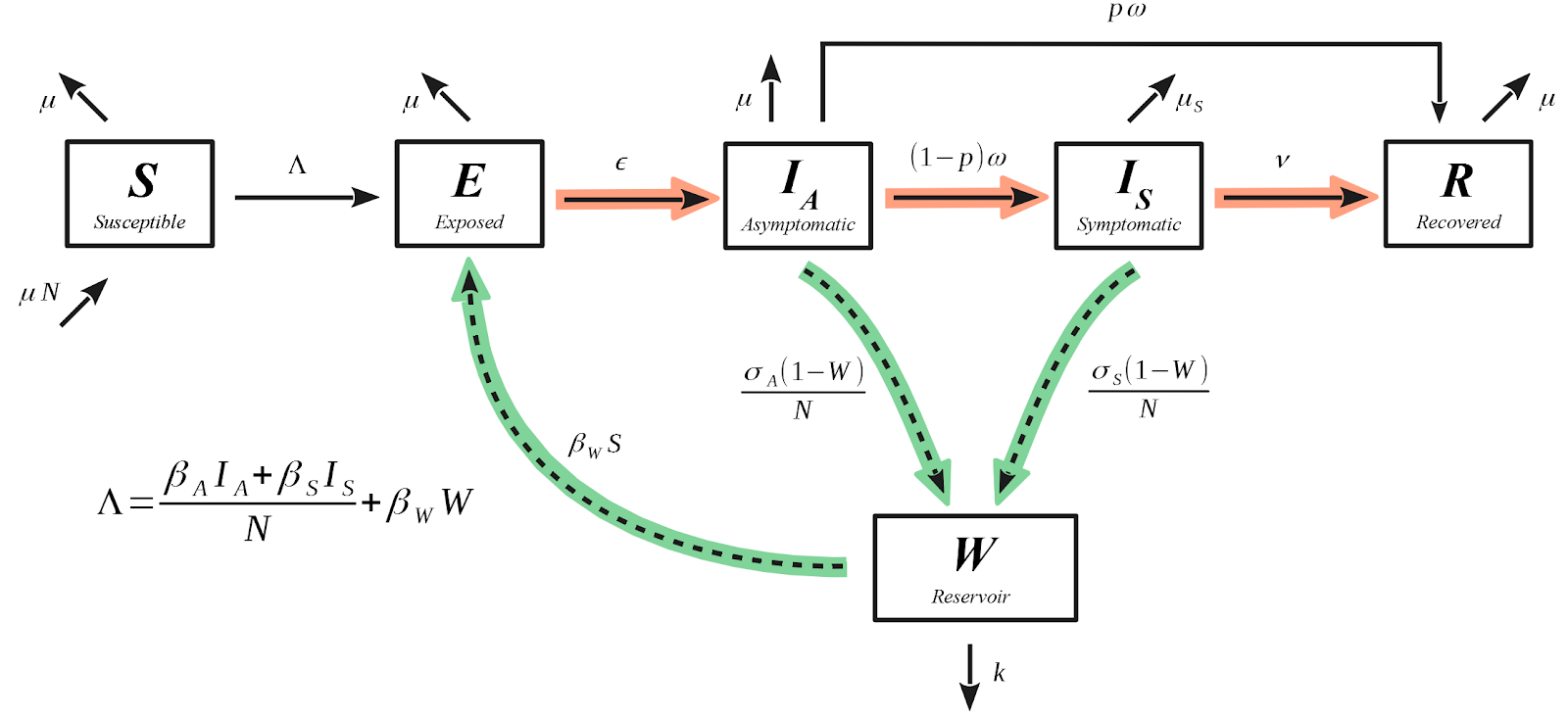


**Fig. S1. SEIR-W model for SARS-CoV-2 transmission**. Compartmental diagram with dynamic information, where the green highlighted arrows represent how the infection couples with the environment, and the red highlighted arrows represent the progression of the infection through individuals. The model is referred to as SEIR-*W* throughout the text.


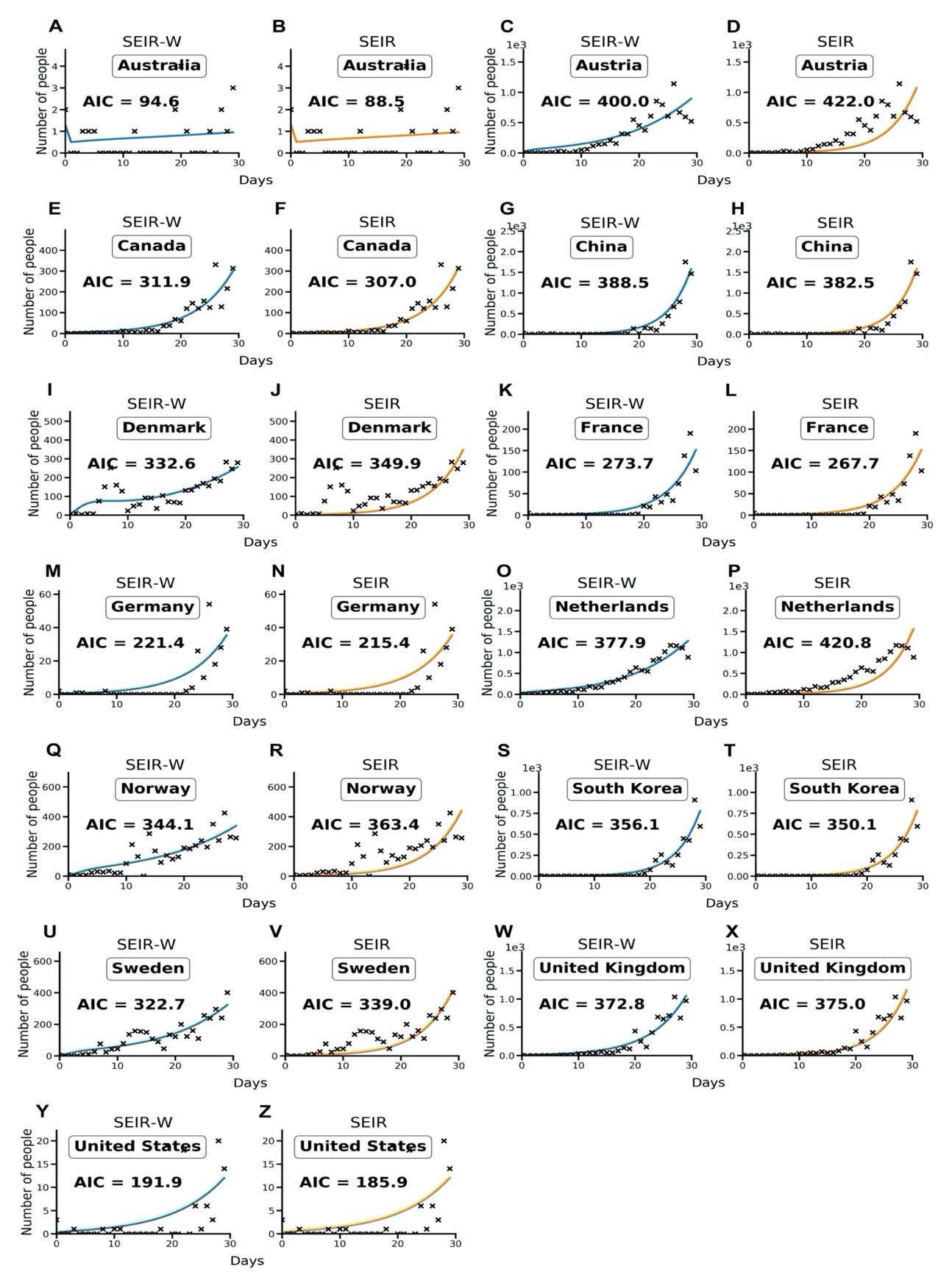


**Fig. S2.** **Graphical depiction of data in the Table S4. A-Z correspond to 13 country fits of the mathematical models—SEIR and SEIR-W**. Four other country fits are depicted in the main text Fig. 1 (Spain, Italy, Iran, Switzerland).

*4. Model sensitivity analysis*

*Partial Rank Correlation Coefficient (PRCC).* A key aspect of model building is a “sensitivity analysis,” or a test of how the model dynamics change as a result of changes in parameters.

We use the *Partial Rank Correlation Coefficient* (PRCC)—an established method—to assess the sensitivity of various aspects of our model with respect to changes in the parameter values of our model [76].

Using this method, we can identify certain parameters which may be quite influential to the dynamics of the infection. Here we briefly review the steps we took to compute the PRCC values for several aspects of the model. As a rough outline of the calculation, one begins by constructing *M* random samples of the parameter values around the predetermined set of expected values of parameters—let us say there are *n* parameters. For each of these *M* samples, which are selected using the *Latin Hypercube Sampling* (LHS) method [77] the value of whatever model aspect is calculated, such as the *ℛ_0_* or the number of people infected after 30 days. In the LHS method, the *n* parameters are varied independently of each other and so this procedure allows us to assess sensitivity of all parameters collectively, rather than assessing the sensitivity of the model with respect to changes in a single parameter at a time. Then the sampled values for each of the *n* parameters are numbered *1* through *M* depending on how they rank in the sample, resulting in *n* vectors of length *M* giving some permutation of the numbers *1* through *M*. The same ranking procedure is performed for the output of the model, whether that is the *ℛ_0_* or something else. Consequently, there are now *n+1* permutations of the numbers *1* through *M*. The next step is to compute the ordinary correlation coefficient between all pairs of these *n+1* vectors, and to arrange them into a matrix *C—*note that *C* is a symmetric matrix. Thus, *C_ij_* gives the correlation between the *ith* and *jth* parameter vector or model value vector. In general, most of these values are expected to be fairly close to 0, except for the diagonal (all ones) and the values associated with correlations with the model value vector. The last step is to compute the matrix inverse of *C*, which we call *B*. Finally, the PRCC values (one for each parameter) are defined by,

$PRCC_{i}=-B_{i n+1}/\sqrt{B_{i i}B_{n+1 n+1}}$ (S8)

where *i* refers to the *ith* parameter.

We compute the PRCC values for four aspects of our model, (1) *ℛ_0_* ,(2) cumulative number of symptomatic infections after 30 days, (3) time to the symptomatic peak (*t_max_*), and (4) symptomatic peak (*I_max_*).

*5. Model robustness to variation in initial fitting parameters*

In order to consider the robustness of the result to various initial parameter values, we examined a range of starting parameter values to begin each iteration of the model-fitting algorithm. As explained in the main text, we originally initialized the fitting parameters (6 parameters in the “with environmental transmission case”, and 3 parameters in the “without environmental transmission case”) to a value of 1.5 (in whatever units are appropriate for each parameter), and allowed the algorithm (Levenberg-Marquardt) to adjust them as needed to locate the best fit to the respective model. The value of 1.5 was chosen because it was expected to be near the true values (best-fit values) of the fitted parameters. In this latest iteration, we expanded the range of starting values to lie within the range 1/2 to 2 times the starting value; i.e. between 0.75 and 3.

One feature that we noticed about the range of fitting results was that for some values of the initial fitting parameters, the fitting algorithm could only locate very poor fits to the data, leading to significantly larger AIC values (Akaike Information Criterion). These poor fits were observed to occur somewhat sporadically across the range of initial fitting parameters sampled and was also observed to vary across the countries sampled. Consequently, in order to address the question of whether the resulting AIC value was lower for one model or the other (i.e. the model *with* or *without* the environmental terms), we had to examine the frequency with which one AIC value was lower than the other across the sampled parameter sweep (0.75 to 3), for each country. Below, we plot a histogram of the frequencies with which the environmental model was observed to have a lower AIC value than the non-environmental model, for each country; i.e. each contribution to the histogram represents the fraction of the initial fitting parameters sampled (between 0.75 and 3) where the AIC for the environmental model was lower than the non-environmental case in one of the countries.


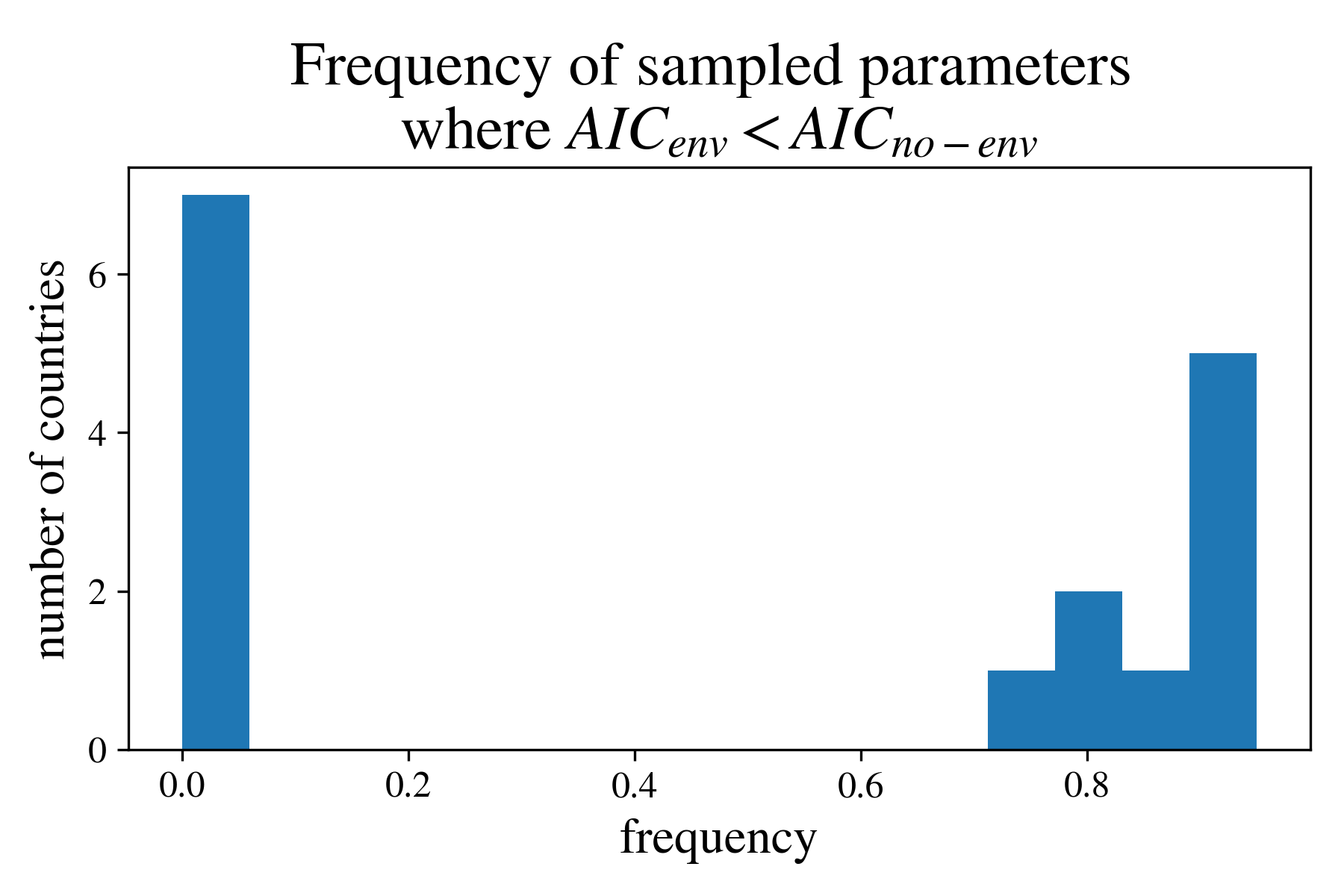


**Figure S3.** **Histogram of the fractions of parameter space where AIC for the environmental model was less than the non-environmental model.** For each country, we compute a fraction of the initial parameter value space (taking values between 0.75 and 3) where the AIC_env was observed to be lower than the AIC_no-env. Each contribution to the histogram represents a single country’s fraction (we refer to the fractions as frequencies).

One finds a bimodal structure to the distribution where in 7 of the countries the AIC for the environmental model was rarely lower than that of the non-environmental case (represented by the single histogram bar near a frequency value of 0), and in 9 of the countries the AIC was consistently lower for the environmental model across the parameter sweep (i.e. AIC_env < AIC_no-env in an excess of 70% of the sampled parameters). Note that there are only a total of 7+9 = 16 countries shown here as the fitting algorithm was unable to locate some of the best-fit parameters for Canada across the entire initial fitting parameter range. Despite missing one country from this dataset, however, the split of 7 to 9 remains consistent with the distribution of results observed for the fits expounded in the main text, where only a starting parameter of 1.5 was used. We therefore expect the results of our fits to be fairly robust, at least for the countries sampled.

|  | **Touch** | **Aerosol** | **Copper** | **Cardboard** | **Stainless steel** | | **Plastic** |
| --- | --- | --- | --- | --- | --- | --- | --- |
| SARS-CoV-2 decay time (1/*k*) |  | 3 hours | 4 hours | 24 hours | 48 hours | 72 hours | |
| *ℛ_0_* |  |  | 2.4 | 2.67 | 2.94 | 3.18 | |
| Time to reach maximum # of *I_S_* | 119 | 91.5 | 88.4 | 65.1 | 56.6 | 52.6 | |
| The maximum number of I_S_ (millions) | 986 | 1 .01 | 1.023 | 1.18 | 1,25 | 1,29 | |
| *The number of I_S_ after 30 days* | 218 | 4376 | 5960 | 58240 | 155390 | 256420 | |
| The number of deaths after 30 days | 2 | 41 | 55 | 461 | 1,133 | 1,814 | |

**Table S5.** **Summary of the “reservoir world” simulations key features**. “Touch” corresponds to the setting where there is no environmental transmission, only person-to-person transmission.

**Supplemental References**

68. Cobey, S. Modeling infectious disease dynamics. Science 368, 713-714 (2020).

69. Lofgren, E. T., Opinion: Mathematical models: A key tool for outbreak response. Proc. Natl. Acad. Sci. 111, 18095–18096 (2014).

70. Central Intelligence Agency. The World Factbook. https://www.cia.gov/library/publications/the-world-factbook/geos/xx.html (2020).

71. Bar-On Y. M., Flamholz, A., Phillips, R. & R. Milo, R. SARS-CoV-2 (COVID-19) by the numbers, 9, 10.7554/eLife.57309 (2020).

72. Russell T. W., et al., Estimating the infection and case fatality ratio for coronavirus disease (COVID-19) using age-adjusted data from the outbreak on the Diamond Princess cruise ship, February 2020. Euro. Surveill. 25, 12; 10.2807/1560-7917.ES.2020.25.12.2000256 (2020).

73. Diekmann, O., Heesterbeek, J. A. P., Roberts, M.G. The construction of next-generation matrices for compartmental epidemic models. J. R. Soc. Interface. 7, 873–885 (2010).

74. Moré, J. J. in Numerical analysis 105–116 (Springer, 1978).

75. Banks, H. R. & Joyner, M. L. AIC under the framework of least squares estimation. Appl. Math. Lett. 74, 33–45 (2017).

76. Hamby, D. M. A review of techniques for parameter sensitivity analysis of environmental models. Environ. Monit. Assess. 32, 135–154 (1994).

77. McKay, M. D., Beckman, R. J. & Conover, W. J. Comparison of three methods for selecting values of input variables in the analysis of output from a computer code. Technometrics. 21, 239–245 (1979).
